# A TROP2-targeting aptamer enables selective drug delivery and potent antitumor activity in pancreatic cancer

**DOI:** 10.1126/sciadv.aee3585

**Published:** 2026-11-11

**Authors:** Yuanding Liu, Zhaofeng Luo, Yuan Gao, Tao Pan, Changjie Yang, Zheng Zhang, Die Yu, Yifan He, Daiquan Chen, Sainan Guo, Yingbin Liu, Yang Sun, Weihong Tan

**Affiliations:** ^1^ Department of Biliary-Pancreatic Surgery, Institute of Molecular Medicine (IMM), Ren Ji Hospital, Shanghai Jiao Tong University School of Medicine, Shanghai 200127, China.; ^2^ Zhejiang Cancer Hospital, Hangzhou Institute of Medicine (HIM), Chinese Academy of Sciences, Hangzhou 310022, China.; ^3^ College of Chemistry and Materials Science, Materials and Chemical Engineering, Shanghai Normal University, Shanghai 200234, China.

## Abstract

Pancreatic ductal adenocarcinoma (PDAC) remains difficult to treat, highlighting the need for strategies that selectively deliver cytotoxic agents to tumor cells. Trophoblast cell-surface antigen 2 (TROP2) has been proposed as a candidate therapeutic target in PDAC, but non-antibody-based delivery formats remain underexplored. Using protein-SELEX, we identified T17, a high-affinity aptamer that specifically binds to TROP2, and conjugated it to monomethyl auristatin E through an MC-Val-Cit-PAB linker to generate the aptamer-drug conjugate T17-M. Preclinical evaluation in cellular and animal models showed that T17-M retained TROP2-dependent binding, underwent clathrin-mediated internalization and lysosomal processing, and suppressed tumor growth in cell-line-derived and patient-derived xenografts. T17-M also reduced liver metastasis and induced calreticulin exposure, ATP release, and HMGB1 secretion, consistent with immunogenic cell death. In immunocompetent models, T17-M promoted dendritic-cell activation and CD8^+^ T cell infiltration, while its combination with PD-1 blockade further enhanced tumor control and prolonged survival. These findings provide preclinical proof-of-concept for T17-M as an alternative TROP2-targeted drug-delivery strategy for PDAC.

## INTRODUCTION

Pancreatic ductal adenocarcinoma (PDAC) is one of the most lethal malignant tumors in human digestive system, and the 5-year survival rate remains approximately 12%. Only 15–20% of PDAC patients have the chance to receive surgical resection, and most patients are already in advanced stages when diagnosed (*1*, *2*). Although having limited effect, cytotoxic chemotherapy backbones, based on FOLFIRINOX (fluorouracil, leucovorin, irinotecan, and oxaliplatin) or gemcitabine plus nab-paclitaxel, remain a primary paradigm in the treatment of PDAC (*3*). Meanwhile, targeted therapy, based on genomics and biomarker expression, provides a new treatment mode for PDAC. Strategies that selectively deliver cytotoxic agents to tumor cells have the potential to improve the therapeutic index by enhancing antitumor efficacy while reducing off-tumor adverse effects (*4*, *5*). However, no therapeutics that deliver cytotoxic agents by targeting cell-surface proteins, such as antibody–drug conjugates (ADCs), have yet been approved for PDAC.

Trophoblast cell-surface antigen 2 (TROP2), encoded by TACSTD2, is a single-pass type I transmembrane glycoprotein originally identified in trophoblasts and physiologically expressed in several epithelial tissues (*6*–*8*). Structurally, TROP2 comprises a glycosylated extracellular domain, a transmembrane region, and a short cytoplasmic tail that can be phosphorylated by protein kinase C. Beyond serving as a surface marker, TROP2 functions as a calcium signal transducer. Phosphorylation and regulated proteolytic activation of TROP2 have been linked to MAPK/ERK, PI3K/AKT, NF-κB, AP-1/cyclin D1, and β-catenin signaling (*9*, *10*). Through these context-dependent networks, aberrant TROP2 expression can promote cell-cycle progression, survival, migration, invasion, stem-like plasticity, and metastatic dissemination (*9*, *10*). Consistent with these functions, TROP2 is frequently overexpressed in breast, lung, gastric, colorectal, ovarian, pancreatic, and other epithelial malignancies, where high expression is often associated with aggressive clinicopathological features and unfavorable survival (*9*–*11*). Its abundant and accessible cell-surface expression, together with its capacity for internalization, has therefore made TROP2 an attractive antigen for targeted drug delivery (*10*).

In PDAC, TROP2 represents a biologically compelling but clinically unresolved therapeutic target. High TROP2 expression was detected in approximately 55% of tumors in a surgical pancreatic cancer cohort and was associated with metastatic disease, higher tumor grade, and shorter progression-free survival (*12*). More recently, a multi-omic analysis of 5,488 PDAC specimens showed that TACSTD2-high tumors were enriched for key driver alterations, including KRAS and TP53, and that high TACSTD2 expression was associated with shorter overall survival, particularly in patients with KRAS-mutant disease (*11*). The clinical success of the TROP2-directed antibody-drug conjugate (ADC) Sacituzumab govitecan, which couples the humanized anti-TROP2 antibody hRS7 to the topoisomerase I inhibitor SN-38, has validated TROP2 as a therapeutically actionable surface antigen in metastatic breast cancer (*13*). However, this success has not yet translated to PDAC. In the phase 1/II IMMU-132-01 basket trial, none of the 16 patients with heavily pretreated PDAC achieved an objective response, with a median progression-free survival of 2.0 months and a median overall survival of 4.5 months (*13*). By contrast, novel TROP2-directed ADCs incorporating more potent payloads, including amanitin, have produced durable tumor regression in preclinical PDAC models, indicating that therapeutic activity depends not only on target expression but also on antigen internalization, linker stability and payload potency (*14*). The dense desmoplastic stroma, heterogeneous antigen distribution, and restricted macromolecular penetration characteristic of PDAC may further constrain conventional antibody-based delivery (*14*). These limitations provide a rationale for developing compact and chemically programmable TROP2-binding aptamers as an alternative targeting modality that may facilitate stromal penetration and enable modular optimization of linker-payload combinations for PDAC.

Aptamers are single-stranded (ss) DNA or RNA oligonucleotides that can fold into unique secondary and tertiary structures and bind their molecular targets with high affinity and specificity (*15*, *16*). Aptamer sequences against diverse molecular targets can be isolated through the systematic evolution of ligands by exponential enrichment (SELEX) and readily modified for diagnostic and therapeutic applications (*17*). Moreover, their ease of chemical synthesis, modularity, and low protein-associated immunogenicity make them attractive ligands for the targeted delivery of cytotoxic agents (*18*, *19*). The use of aptamers as targeting ligands for cancer cells has therefore become an increasingly explored strategy in drug design (*20*–*22*). Compared with antibody–drug conjugates (ADCs), aptamer–drug conjugates (ApDCs) may offer distinct pharmacological properties that are particularly relevant to PDAC. Their smaller molecular size may facilitate tissue penetration and intratumoral distribution. In addition, ApDCs lack Fc-mediated effector functions and may have lower protein-associated immunogenicity than antibody-based agents. These features support the investigation of ApDCs as an alternative platform for targeted drug delivery in PDAC.

In this study, we evaluated TROP2 as a candidate cell-surface target in PDAC. Using protein-SELEX followed by in vitro and in vivo validation, we identified a high-affinity TROP2-binding ssDNA aptamer. We then generated the aptamer–drug conjugate T17-M by coupling the aptamer to monomethyl auristatin E through an MC–Val–Cit–PAB linker. T17-M underwent clathrin-mediated endocytosis and lysosomal processing, thereby enabling intracellular payload release and inducing apoptosis. It also showed antitumor activity in multiple PDAC models. Beyond its direct cytotoxic activity, T17-M induced immunogenic cell death, as indicated by calreticulin exposure, ATP release, and HMGB1 secretion, and promoted dendritic cell activation and CD8^+^ T cell infiltration. Moreover, combination with PD-1 blockade further enhanced tumor control and prolonged survival. Together, these findings provide preclinical proof of concept for T17-M as an alternative TROP2-targeted drug-delivery strategy for PDAC and support its further evaluation in combination-treatment settings.

## RESULTS

### TROP2 is a promising ApDC target in PDAC

We first examined the expression of TACSTD2, which encodes TROP2, across digestive system malignancies using the GEPIA database (*23*). TACSTD2 was evidently upregulated in five of the seven analyzed digestive system tumor types compared with the corresponding normal tissues, with particularly high expression in pancreatic ductal adenocarcinoma (PDAC) (Fig. 1A and fig. S1A). Moreover, according to the TCGA database, the high expression level of TACSTD2 was correlated with poor PFI (Progression Free Interval, *P* = 0.00076) and OS (Overall Survival, *P* = 0.047) in PDAC patients (Fig. 1B). Besides, gene set enrichment analysis (GSEA) revealed a panel of signaling pathways related with tumorigenesis and tumor progression, were enriched in transcriptional profiles of PDAC with higher TACSTD2 expression (fig. S1B).

**Fig. 1.**
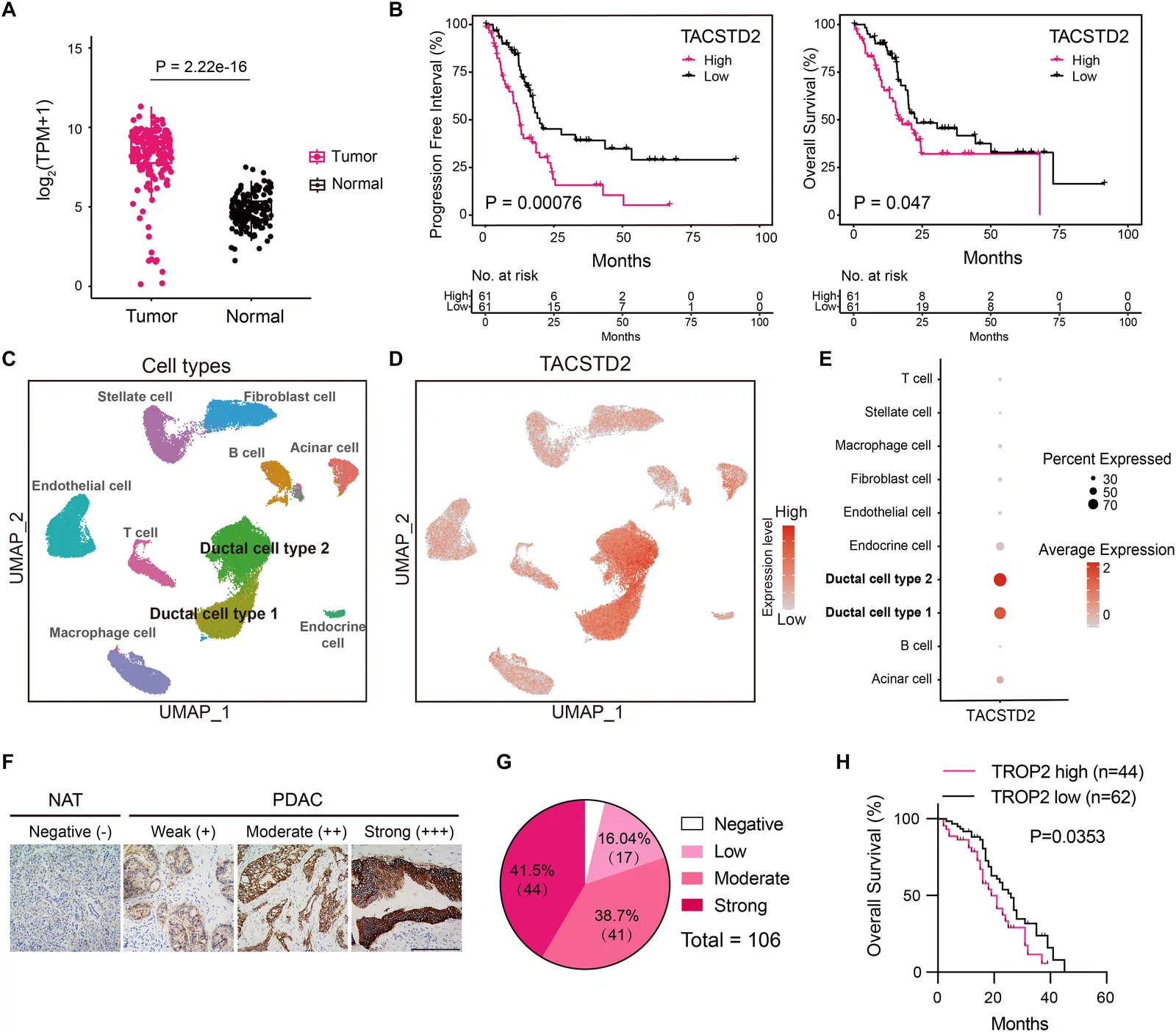
TROP2 is a promising ApDC target in PDAC. (**A**) Expression level of TACSTD2 in PDAC and pancreatic normal tissue. Statistical significance was determined using a two-sided Wilcoxon rank-sum test. (**B**) The Kaplan–Meier survival curves for progression-free interval (PFI) and overall survival (OS) in the TCGA-PAAD cohort stratified by TACSTD2 expression (high, *n* = 61; low, *n* = 61). *P* values were determined using two-sided log-rank tests. (**C** to **E**) Single-cell RNA-seq analysis of 24 PDAC and 11 adjacent normal samples. (C) UMAP showing major cell types. (D) UMAP feature plot of TACSTD2 expression. (E) Dot plot summarizing TACSTD2 across cell populations (dot size = percent expressed; color = scaled average expression). (**F**) Representative IHC images of TROP2 in non-tumor adjacent tissue (NAT) and PDAC, scored as negative (−), weak (+), moderate (++), or strong (+++). Scale bar, 200 μm. (**G**) Distribution of TROP2 IHC staining scores in 106 PDAC samples. (**H**) The Kaplan–Meier survival curves for overall survival (OS) in the PDAC-RJ cohort stratified by TROP2 IHC. TROP2-high was defined as strong (+++) staining (*n* = 44), whereas TROP2-low included negative (−), weak (+), and moderate (++) staining (*n* = 62). The *P* value was determined using a two-sided log-rank test. Exact *P* values are shown in the corresponding panels.

To determine the cellular source of TACSTD2 expression in PDAC, we analyzed a single-cell RNA-sequencing dataset comprising 24 PDAC specimens and 11 normal samples (*24*). The results suggested that TACSTD2 was upregulated in malignant ductal cells in PDAC, but not other cell types such as fibroblast cell, immune cell or endothelial cell. Also, ductal cell type 2, which possessed more malignant degree than ductal cell type 1, exhibited higher expression level (Fig. 1, C to E). To further validate and determine the protein expression level of TROP2, we carried out immunohistochemistry (IHC) staining of a cohort containing 106 patients tumor regions and 10 paired nontumor adjacent tissue (NAT), which had been pathologically confirmed by Department of Pathology, Renji Hospital. TROP2 staining was positive in 102 of 106 tumors (96.2%), whereas staining in NAT samples was substantially lower. TROP2 expression was evaluated as low, medium and high in 16.04%, 38.7% and 41.5% respectively, which were consistent with previous reports (Fig. 1, F and G) (*8*, *12*). For exploratory clinical stratification, strong (+++) staining was defined as TROP2-high, identifying 44 of 106 patients (41.5%), whereas negative, weak, and moderate staining were grouped as TROP2-low. Patients in the TROP2-high group had notably shorter overall survival than those in the TROP2-low group (Fig. 1H).

Consistent with the tissue-level findings, CCLE analysis and western blotting identified BxPC-3 and Capan-2 as TROP2-high PDAC cell lines (fig. S1, C and D) (*25*, *26*). Immunofluorescence further demonstrated prominent membrane localization of TROP2 in BxPC-3 and Capan-2 cells, whereas no signal was detected in the nonmalignant pancreatic ductal cell line HPNE (fig. S1E). Together, we confirmed that TROP2 was widely overexpressed on the cell surface of PDAC tumor cells rather than normal cells or other adjacent tissues, and it was rational for TROP2 to be a therapeutic target in PDAC.

### The SELEX and characterization of TROP2 aptamer

Aptamers, developed in 1990, are single-stranded (ss) DNA or RNA which can be isolated and identified by (SELEX) process (Fig. 2A), and can bind targets specifically and tightly. We performed SELEX procedure with recombinant TROP2 protein that contains extracellular domain (His27-Thr274), and with 8 rounds SELEX and subsequently sequencing, two potential aptamers TROP2–1 and TROP2–17 were selected for further study (table S1). The secondary structures of these two aptamers were predicted by the RNAfold webserver and the tertiary structures were predicted using RNAComposer (*27*, *28*). Obviously, TROP2–1 and TROP2–17 contained spiral or clover secondary structure respectively, which was classical structure of aptamer (Fig. 2B and fig. S2A) (*29*).

**Fig. 2.**
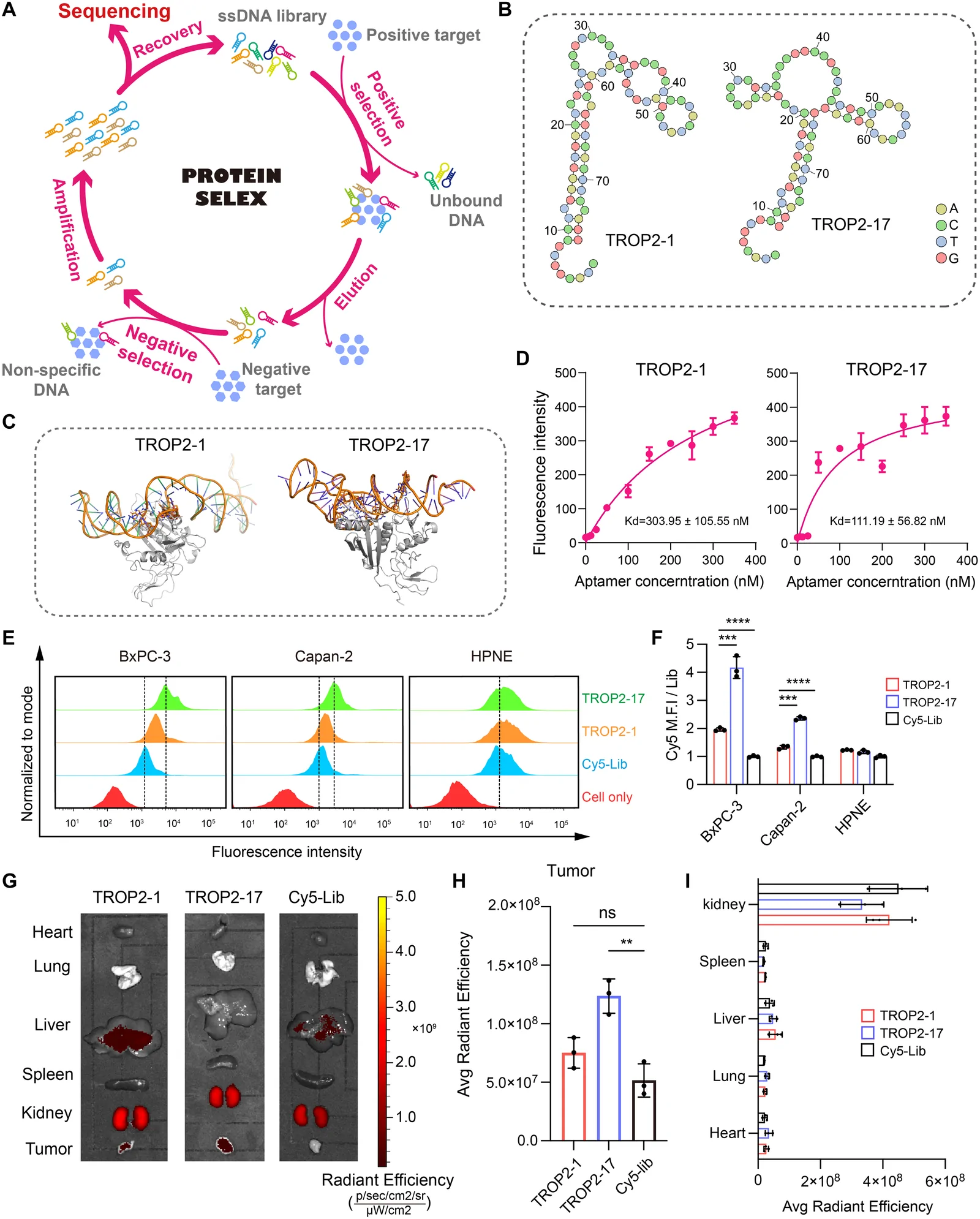
The SELEX and characterization of TROP2 aptamer. (**A**) Schematic of the protein SELEX procedure for TROP2-targeting aptamer selection, including positive selection, negative selection, amplification, and next-generation sequencing. (**B**) Predicted secondary structures of TROP2–1 aptamer and TROP2–17 aptamer. Nucleotides are color coded as indicated. (**C**) Molecular docking models of TROP2–1 and TROP2–17 with the TROP2 extracellular domain (ECD). (**D**) The dissociation constant measurement of TROP2–1 and TROP2–17 by using negatively charged polystyrene microspheres and analyzed by flow cytometry. Data are presented as means ± SD (n = 3 independent measurements). (**E** and **F**) Flow cytometric analysis of Cy5-labeled aptamer binding to TROP2-high BxPC-3 and Capan-2 cells and nonmalignant HPNE cells. (E) Representative flow cytometry histograms. (F) Quantification of median fluorescence intensity (MFI), normalized to the Cy5-Lib control. (**G**) Ex vivo fluorescence images of tumors and major organs from BxPC-3 CDX mice at 2 hours post-injection of TROP2–1, TROP2–17, or Cy5-Lib. Color bar indicating radiant efficiency. (**H** and **I**) Quantification of average radiant efficiency in tumors (H) and the indicated major organs (I). Data in (F), (H), and (I) are presented as means ± SD (*n* = 3 independent experiments for F and *n* = 3 mice per group for H and I). Statistical significance in (F) was determined using two-way ANOVA followed by Tukey’s multiple-comparisons test. Statistical significance in (H) was determined using one-way ANOVA followed by Tukey’s multiple-comparisons test. ns, not significant; *P < 0.05, **P < 0.01, ***P < 0.001, and ****P < 0.0001.

To determine which aptamer is a better one, we performed a series of experiments to assess TROP2–1 and TROP2–17 from several aspects. First, we conducted molecular dynamics simulations (MDS) (*30*, *31*) between the aptamers and TROP2 extracellular domain. We found only TROP2–17 could form a three-loop-stem structure and the binding energy of TROP2–1 and TROP2–17 was −37.64 ± 1.09 and −56.39 ± 1.26 (kJ/mol) respectively. The detail of the binding between the protein residues and aptamers’ bases were summarized in table S2 (Fig. 2C and fig. S2, B and C). In the dissociation constant (Kd) measurement, by incubating different concentrations of Cy5-labeled aptamers with negatively charged polystyrene microspheres, which could adsorb the recombinant TROP2 protein extracellular domain strongly on the surface (*32*), we found the Kd value of TROP2-1 and TROP2–17 was 303.95 ± 105.55 nM and 111.19 ± 56.82 nM respectively (Fig. 2D).

Subsequently, to evaluate these two aptamers binding with the cancer cells, Cy5-lib, which was a random sequence, Cy5-labeled TROP2–1 and TROP2–17 were incubated with BxPC-3, Capan-2, as TROP2-positive control, and HPNE, as TROP2-negative control, respectively. Cy5-labeled TROP2–1 and TROP2–17 both bound TROP2-high BxPC-3 and Capan-2 cells, whereas minimal binding was detected in HPNE cells (Fig. 2, E and F). TROP2–17 produced a greater fluorescence shift than TROP2–1 in both PDAC cell lines. Moreover, siRNA-mediated TROP2 knockdown markedly reduced TROP2–17 binding, confirming that its cellular association was TROP2 dependent (fig. S3).

Additionally, to compare the in vivo tumor-targeting properties of TROP2–1 and TROP2–17, we injected Cy5 labeled aptamers into the BxPC-3 cell derived xenograft (CDX) and, after 2 hours, the tumors and major organs were visualized by in vivo imaging system (IVIS). As shown in Fig. 2, G to I, the fluorescence of TROP2–17 could accumulate in tumors more efficiently compared with Cy5-Lib and TROP2–1 group, and the results also indicated kidney was the major metabolism organ for TROP2–1 and TROP2–17.

In conclusion, through SELEX process, we obtained two potential candidate aptamers, and depending on the evaluation of different aspects, include protein, cell and in vivo, we determined TROP2–17 was more excellent than TROP2–1.

### Design, construction, and functional characterization of TROP2 targeting ApDC

As aforementioned, TROP2–17 (T17) reveals excellent TROP2 targeting efficiency. It was employed in the construction of ApDC by conjugating T17 with MC-Vc-Pab-MMAE (VC-MMAE), an effective ADC linker-warhead combination that has been used in clinical practice (*4*, *33*, *34*) (Fig. 3A). Mass spectrometry was carried out to characterize the successful conjugation of T17 and MC-Vc-Pab-MMAE (T17-M). Flow cytometry was used to evaluate its binding ability to PDAC cells (T17-M-Cy5). The results showed no discrepancy in binding ability between T17 and T17-M on the cellular level (Fig. 3B).

**Fig. 3.**
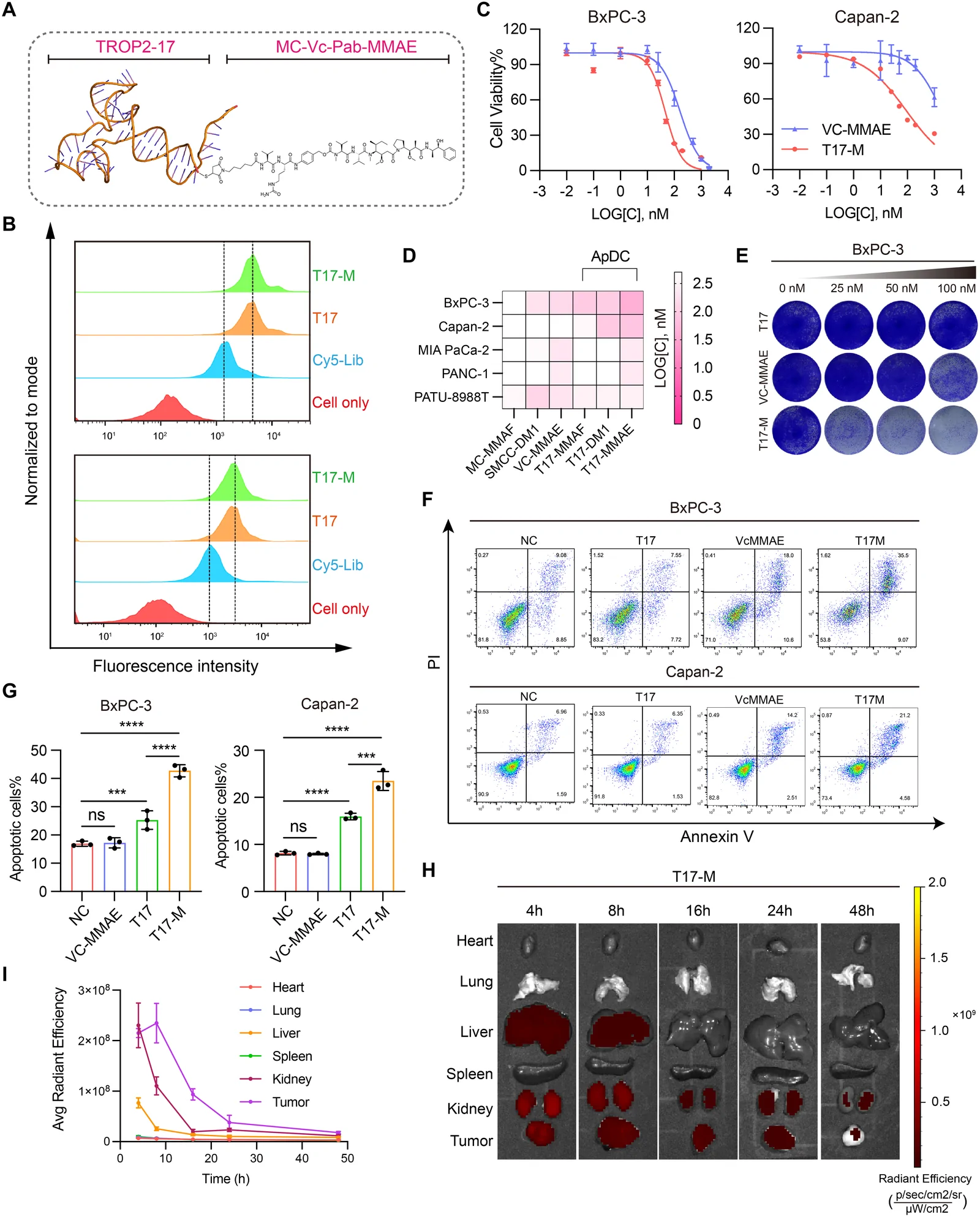
Design, construction, and functional characterization of TROP2 targeting ApDC. (**A**) The design diagram of the ApDC composed of the TROP2–17 aptamer (T17) conjugated to monomethyl auristatin E (MMAE) via an MC–Val–Cit–PAB linker (T17-M). (**B**) Representative flow cytometry histograms showing the binding of Cy5-labeled T17-M and T17 to BxPC-3 and Capan-2 cells. Cy5-Lib and untreated cells were used as controls. (**C**) Dose–response curves of VC-MMAE versus T17-M in BxPC-3 and Capan-2 cells measured by CCK-8. The cells were treated with gradient concentration of VC-MMAE or T17-M for 72 h. (**D**) Heatmap summarizing the cytotoxic potency (log IC_50_) of the indicated ApDC constructs across pancreatic cancer cell lines. (**E**) Long-term cell proliferation assay (stained by crystal violet) of BxPC-3 after the indicated treatments and concentrations. (**F**) annexin V/PI flow cytometry of apoptosis in BxPC-3 and Capan-2 following 24 h exposure to T17, VC-MMAE, or T17-M. (**G**) Quantification of apoptotic cells in (F). (**H**) Representative ex vivo fluorescence images showing the time-dependent biodistribution of Cy5-T17-M in tumors and major organs (heart, lung, liver, spleen, kidney) of BxPC-3 CDX mice at 4, 8, 16, 24, and 48 h post-injection. The color scale indicates radiant efficiency. (**I**) Quantification of average radiant efficiency in tumors and the indicated organs over time. Data in (C) and (G) are means ± SD from three independent experiments; data in (I) are means ± SD (*n* = 3 mice per time point). Statistical significance in (G) was assessed separately for each cell line using one-way ANOVA followed by Tukey’s multiple-comparisons test. ns, not significant; *P < 0.05, **P < 0.01, ***P < 0.001, and ****P < 0.0001.

Next, the anti-tumor ability of ApDC was explored. The IC_50_ of T17-M was ranging from 39.8 to 54.04 nM and 72.08 to 118.1 nM, which was 2.85- to 14.45-fold lower than the IC_50_ of VC-MMAE in BxPC-3 and Capan-2 cells (Fig. 3C). In contrast, the difference between T17-M and VC-MMAE was attenuated in the lower-TROP2 PANC-1, PATU-8988 T, and MIA PaCa-2 cell lines (fig. S4). Comparative testing of T17 conjugates carrying MMAE, DM1, or MMAF indicated that the MMAE-containing T17-M construct had the most favorable overall cytotoxicity profile across the tested pancreatic cancer cell lines (Fig. 3D and fig. S5 and S6). Long-term cell proliferation assay showed that T17-M had a better inhibitory effect than VC-MMAE on cell proliferation (Fig. 3E and fig. S7). Through apoptosis assay, we found the incubation of T17-M induced more cell apoptosis in both cell lines (Fig. 3, F and G). In addition, cell-cycle analysis showed that T17-M induced G2/M-phase accumulation, consistent with the antimitotic mechanism of MMAE (fig. S8) (*35*). Despite we confirmed that the ability of T17-M binding cells was parallel to mono aptamer T17 in vitro, we were not clear about its behavior in vivo, so we examined the organ distribution of T17-M-Cy5 in BxPC-3 and Capan-2 CDX models (Fig. 3, H and I, and fig. S9). The results revealed that T17-M showed significant targeting ability and temporarily accumulated in liver no more than 8 hours and was metabolized by kidneys mainly.

### The internalization efficiency and mechanism of ApDC

As a tubulin inhibitor, MMAE acts intracellularly, we next examined the internalization of T17-M. Confocal microscopy demonstrated clear uptake of Cy5-labeled T17-M by cells in two independent patient-derived PDAC organoids (Fig. 4, A and B). Meanwhile, at the cellular level, stronger fluorescence could be detected when T17-M was incubated with TROP2-positive PDAC cells than with TROP2-negative PDAC cells or HPNE (fig. S10). To further identify the internalization mechanism by which T17-M entered the cells, variety endocytic pathway associated inhibitors were used. Results show that when using Dynasore, which is an inhibitor of clathrin-dependent endocytosis, the intensity of fluorescence was incredibly decreased in both cell lines. However, when using other inhibitors, such as the caveolae-mediated endocytosis inhibitor Genistein, the adenosine triphosphatase inhibitor Sodium orthovanadate, macropinocytosis inhibitor wortmannin and lipid raft inhibitors Nystatin (*36*–*38*), almost had no obvious influence on the absorption of T17-M (Fig. 4, C and D, and fig. S11). These findings indicate that T17-M uptake occurs predominantly through a clathrin-associated endocytic pathway.

**Fig. 4.**
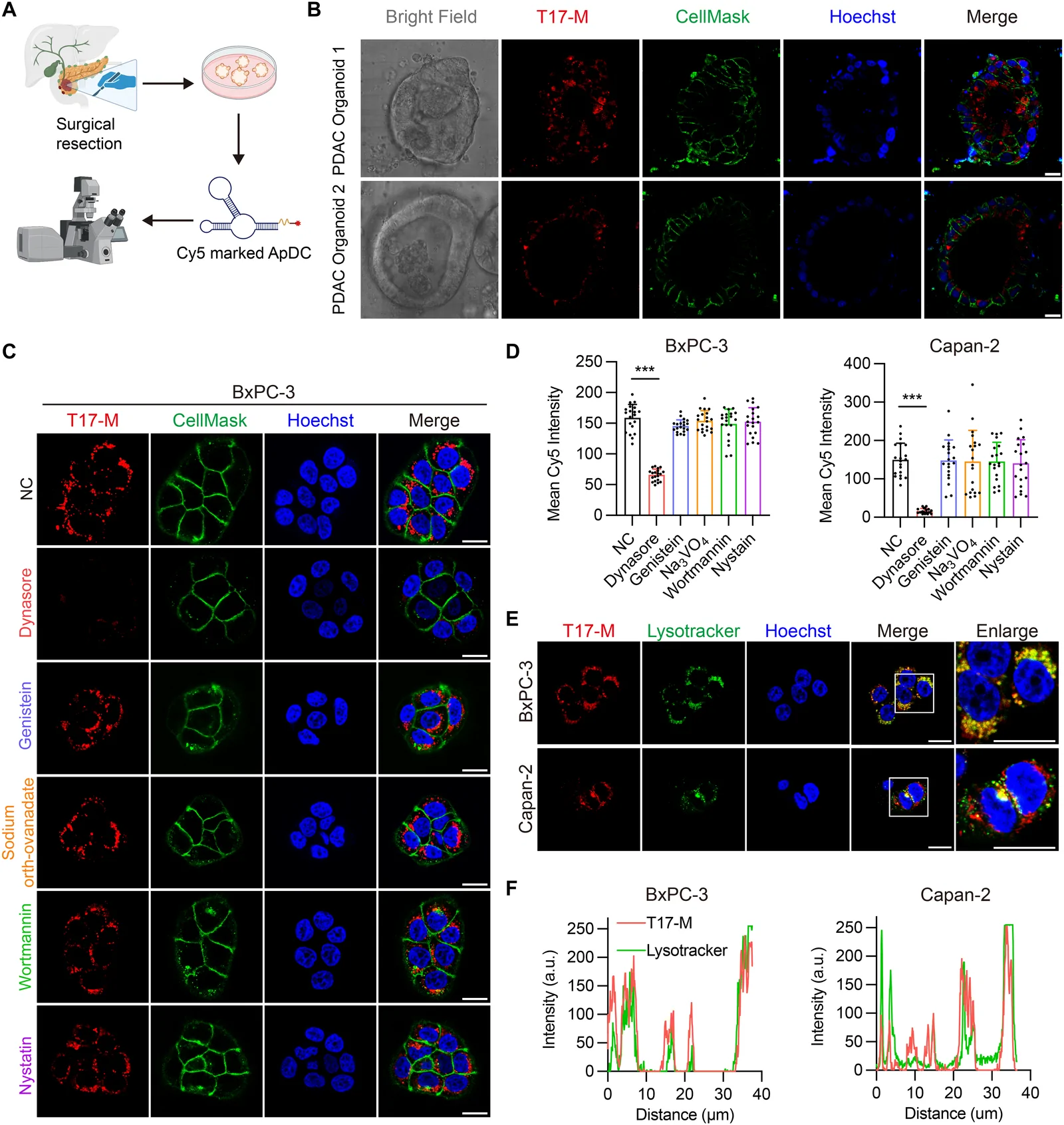
The internalization efficiency and mechanism of ApDC. (**A**) Workflow of the imaging of patient derived PDAC organoids were incubated with Cy5 labeled ApDC and imaged by confocal microscopy. The figure was created using BioRender. 陈, 酯. (2026) https://BioRender.com/bfytqdm. (**B**) Confocal images showing internalization of Cy5 labeled T17-M (red) in two independent PDAC organoids; CellMask showing membranes (green) and Hoechst showing nuclei (blue). (**C**) Effects of endocytic inhibitors on T17-M uptake in BxPC-3 cells. Cells were pretreated with dynasore, genistein, sodium orthovanadate, wortmannin or nystatin. (**D**) Quantification of mean Cy5 fluorescence intensity per cell in BxPC-3 (left) and Capan-2 (right). (**E**) Representative colocalization of internalized T17-M (red) with lysosomes labeled by LysoTracker (green). (**F**) Line-scan profiles illustrating spatial colocalization between T17-M and LysoTracker signals. Scale bars, 20 μm. Data in (D) are presented as means ± SD. Statistical significance was assessed separately for each cell line using one-way ANOVA followed by Dunnett’s multiple-comparisons test, with each inhibitor group compared with the untreated control. ***P < 0.001.

Previous studies have been proved that MC-VC-Pab-MMAE is equipped with a linker which can be cleaved by cathepsin B proteases in cell endosomes/lysosomes but can stay stable under physiological conditions, which means T17-M entered into the cells have to liberate the toxic drug through lysosomes (*34*, *39*–*41*). Thus, we explored the colocalization of lysosomes and T17-M-Cy5 with LysoTracker. The results revealed T17-M-Cy5 possessed good colocalization with lysosome and the Pearson’s coefficient between LysoTracker and T17-M-Cy5 was calculated to be 0.84 in BxPC-3 and 0.66 in Capan-2 (Fig. 4, E and F). PAGE analysis further demonstrated pH- and cathepsin B–dependent processing of T17-M (fig. S12). Prominent shorter nucleic-acid bands appeared only at pH 5.0 in the presence of cathepsin B, indicating linker cleavage and release of the aptamer fragment, and E-64, which is a cysteine-protease inhibitor, abrogated this effect. Minimal degradation occurred at pH 7.0, demonstrating serum-like stability. To sum up, the ApDC T17-M selectively internalizes into TROP2-expressing cells via clathrin-mediated endocytosis, traffics to acidic lysosomal compartments, and undergoes cathepsin B–dependent linker cleavage, thereby achieving controlled and tumor-specific drug release.

### In vivo antitumor efficacy of T17-M in PDAC xenograft and metastasis models

Before the anti-tumor activity tests in vivo, to ensure the safety of T17-M, we inspected the systemic toxicology of T17-M in ICR mice. Complete blood counts, liver and kidney function index, and endpoint body weights were assessed at 1, 14, and 28 days after intravenous administration. No significant differences were detected between T17-M-treated mice and untreated healthy controls at the tested dose (fig. S13, A to C). The H&E staining of the major organ (heart, lung, liver, spleen, and kidney) revealed no obvious variation between healthy and treated group (fig. S13D), consistent with the blood results. Repeated-dose safety was subsequently evaluated in Sprague–Dawley rats administered saline or low, middle, or high doses of T17-M once every 4 days for four cycles (q4d × 4) (fig. S14A). No evident dose-dependent body-weight loss was observed, and most hematologic parameters remained comparable among the treatment groups (fig. S14, B and C). In contrast, the high-dose group exhibited marked elevations in alanine aminotransferase (ALT), aspartate aminotransferase (AST), alkaline phosphatase (ALP), γ-glutamyl transferase (γ-GT), and total bile acids, suggesting a potential hepatobiliary toxicity signal at high systemic exposure (fig. S14D). Despite these biochemical changes, no overt histopathologic abnormalities were observed in the examined major organs (fig. S15). Pharmacokinetic analysis showed that the plasma AUC of free MMAE accounted for only 3.83 ± 0.42% of the total MMAE AUC, indicating limited systemic exposure to unconjugated payload (fig. S16A). Tissue-distribution analysis revealed that MMAE exposure was highest in the kidney, followed by the liver, with substantially lower levels detected in the other examined organs (fig. S16B). Together, these findings indicate that T17-M was generally well tolerated at the therapeutic dose, whereas high-dose exposure was associated with a potential, dose-limiting hepatobiliary safety signal.

We next evaluated antitumor efficacy in BxPC-3 and Capan-2 CDX models (Fig. 5A). T17-M produced the greatest suppression of tumor growth in both models compared with DPBS, T17, and VC-MMAE (Fig. 5, B and C). Ki-67 immunohistochemistry confirmed a marked reduction in proliferating tumor cells after T17-M treatment (Fig. 5, D and E). Thus, conjugation of MMAE to the TROP2-targeting aptamer enhanced antitumor activity relative to the corresponding unconjugated linker-payload.

**Fig. 5.**
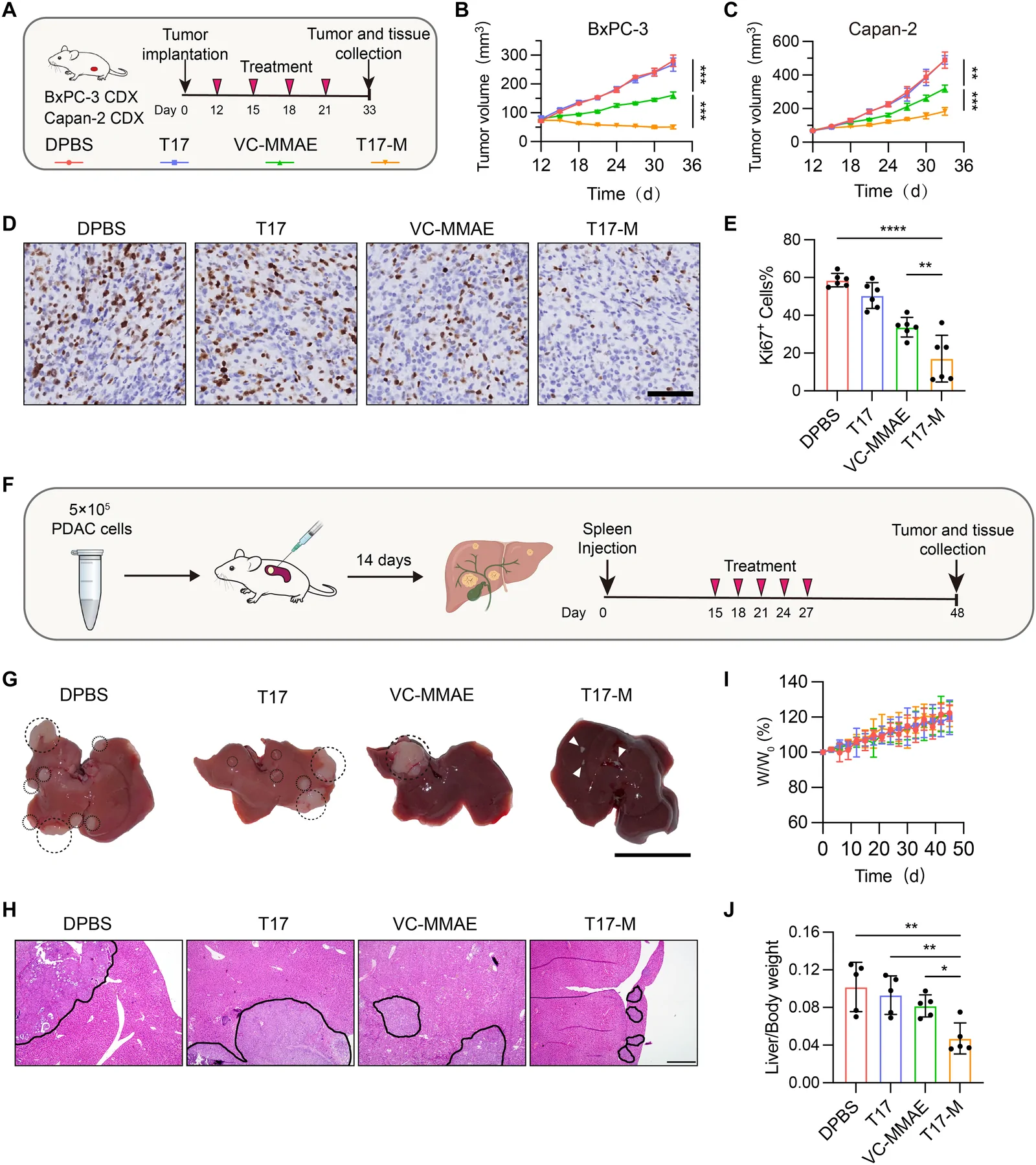
In vivo antitumor efficacy of T17-M in PDAC xenograft and metastasis models. (**A**) Schematic of the experimental design. BxPC-3 and Capan-2 CDX models were established, and mice were treated with DPBS, T17, VC-MMAE, or T17-M at the indicated time points. (**B** and **C**) Tumor growth curves of BxPC-3 (B) and Capan-2 (C) CDX under the indicated treatments. (**D** and **E**) Representative Ki-67 IHC staining of BxPC-3 tumor sections (D) and quantification of Ki-67^+^ tumor cells (E). (**F**) Schematic of the experimental design. The PDAC liver metastasis model generated via splenic injection of tumor cells, followed by indicated treatments. The figure was created using BioRender. 陈, 酯 (2026) https://BioRender.com/ac37gv4. (**G**) Representative macroscopic images of liver metastases under the indicated treatments; dashed circles denote metastatic nodules. Scale bar, 1 cm. (**H**) Representative H&E staining of liver sections showing metastatic lesions. Scale bar, 200 μm. (**I**) Body-weight changes normalized to the corresponding baseline values. (**J**) Liver-to-body weight ratios. Data are presented as means ± SD (*n *= 5 mice per group). Tumor volumes at the final experimental time point in (B) and (C) were analyzed using one-way ANOVA followed by Tukey’s multiple-comparisons test. Statistical significance in (E) and (J) was assessed using one-way ANOVA followed by Tukey’s multiple-comparisons test. ns, not significant; *P < 0.05, **P < 0.01, ***P < 0.001, and ****P < 0.0001.

As one of the most aggressive malignancies, PDAC tend to metastasize preferentially to the liver at early stage (*2*, *42*–*44*). Gene Ontology analysis of TACSTD2 associated genes identified several enriched biological processes related to cell migration and metastasis (fig. S17). In vitro, T17-M markedly reduced the migration of BxPC-3 and Capan-2 cells compared with the control, T17, and VC-MMAE groups (fig. S18). The antimetastatic activity of T17-M was then evaluated in a liver-metastasis model generated by splenic injection of PDAC cells (Fig. 5F). T17-M treated mice displayed fewer and smaller macroscopic liver lesions and reduced metastatic areas on histologic examination (Fig. 5, G and H). No substantial differences in normalized body weight were observed among the treatment groups (Fig. 5I), whereas the liver-to-body weight ratio was notably reduced in the T17-M group (Fig. 5J). Collectively, these results demonstrate that T17-M suppresses both primary PDAC tumor growth and hepatic metastatic burden.

### Tumor targeting and antitumor efficacy of T17-M in PDAC PDX model

To evaluate T17-M in a clinically relevant context, we employed a patient-derived xenograft (PDX) model established from surgical tumor fragments that retain components of the tumor microenvironment, including extracellular matrix, cancer-associated fibroblasts, and vasculature (Fig. 6A). Firstly, the distribution of T17-Cy5 and T17-M-Cy5 in PDX models was evaluated, results revealed similar targeting property to other mice models (Fig. 6, B to D), indicating that conjugation to MMAE did not compromise TROP2 targeted delivery. In contrast, the non-targeting Lib-M control showed minimal tumor accumulation, further validating the target-specific delivery capability of T17-M (fig. S19). T17-M produced the strongest antitumor activity in the PDX model. Excised tumors were visibly smaller after T17-M treatment (Fig. 6E), tumor growth was markedly delayed over time (Fig. 6F), and endpoint tumor weights were markedly lower than all controls including the group of VC-MMAE (Fig. 6G). Consistent with these volumetric results, tumors of T17-M group displayed a pronounced decrease in proliferative index by Ki-67 immunohistochemistry (Fig. 6H) with evidently fewer Ki-67^+^ cells (Fig. 6I). Collectively, these data demonstrate that T17-M preserves TROP2 mediated tumor targeting while translating it into superior antitumor efficacy in a clinically relevant PDX setting.

**Fig. 6.**
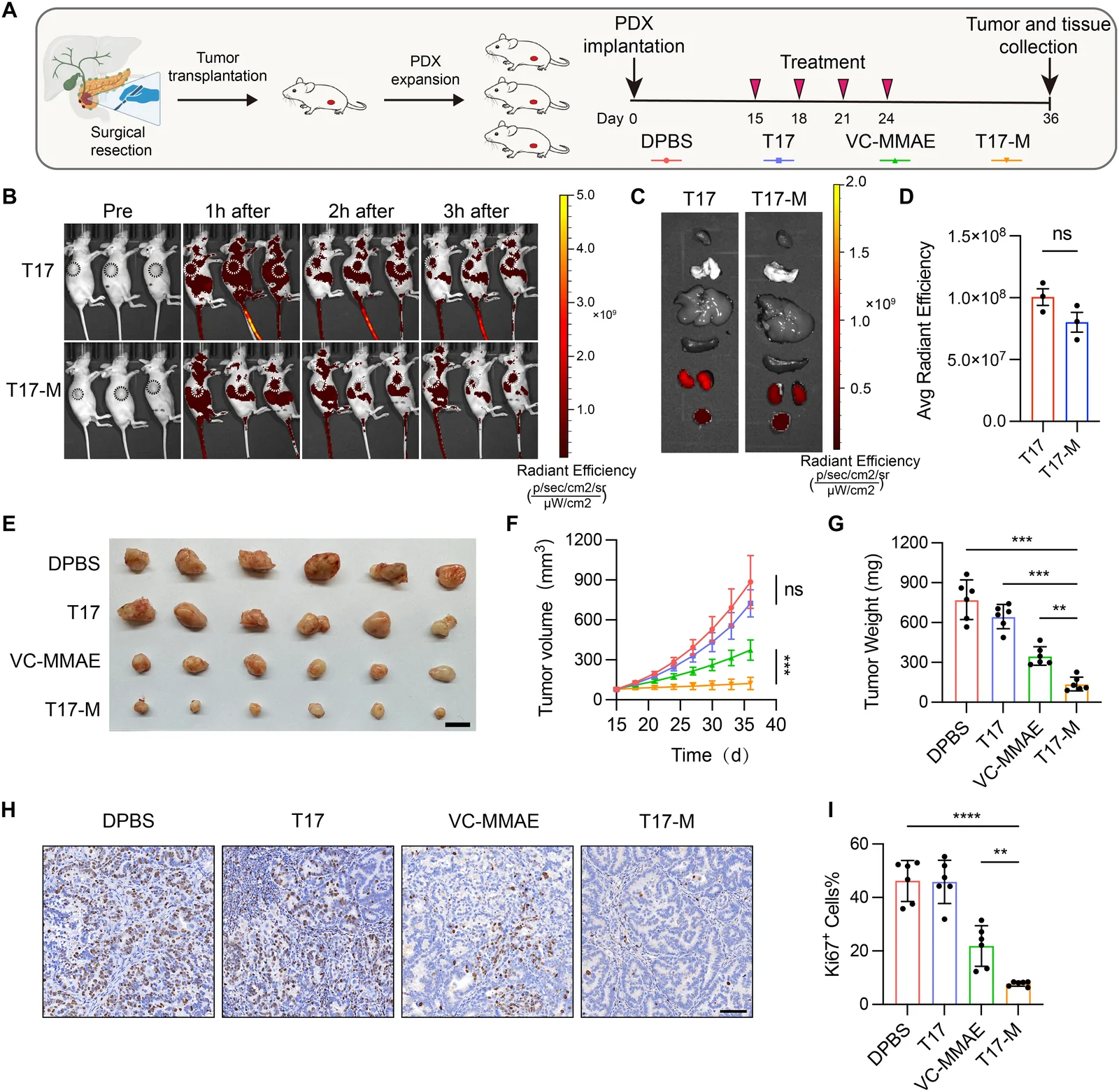
Tumor targeting and antitumor efficacy of T17-M in PDAC PDX model. (**A**) Schematic of the experimental design. Fresh PDAC tissues were expanded in mice and re-implanted, followed by systemic treatments at the indicated time points. The figure was created using BioRender. 陈, 酯 (2026) https://BioRender.com/ul9r5uo. (**B**) In vivo fluorescence images showing whole-body fluorescence after injection of Cy5-labeled T17 or T17-M at baseline (Pre) and 1, 2, and 3 hours post-injection. Color bars indicate radiant efficiency. (**C**) Ex vivo fluorescence images of major organs and tumors 3 hours after injection of T17 or T17-M. Color scales indicate radiant efficiency. (**D**) Quantification of average radiant efficiency in tumors. (**E**) Images of excised tumors from the indicated groups. Scale bar, 1 cm. (**F**) Tumor growth curves under the indicated treatments. (**G**) Final tumor weights at endpoint for each group. (**H**) Representative Ki-67 IHC staining of PDX tumor sections. Scale bar, 100 μm. (**I**) Quantification of Ki-67^+^ tumor cells across treatment groups**.** Data are presented as means ± SD (*n *= 3 mice per group for D and *n *= 6 mice per group for F, G, and I). Statistical significance in (D) was determined using a two-tailed unpaired Student’s *t* test. Tumor volumes at the final experimental time point in (F) and data in (G) and (I) were analyzed using one-way ANOVA followed by Tukey’s multiple-comparisons test. ns, not significant; *P < 0.05, **P < 0.01, ***P < 0.001, and ****P < 0.0001.

### T17-M induces immunogenic cell death and remodels tumor immune microenvironment

MMAE has been reported to induce immunogenic cell death (ICD) in tumor cells (*45*, *46*). We therefore investigated whether T17-M elicited ICD in PDAC cells. Confocal microscopy demonstrated prominent cell-surface calreticulin exposure after T17-M treatment, which was confirmed by flow cytometry in BxPC-3 and Capan-2 cells (Fig. 7, A and B). T17-M also increased extracellular ATP release and HMGB1 secretion in both cell lines (Fig. 7, C and D). These findings show that T17-M induces multiple hallmarks of ICD.

**Fig. 7.**
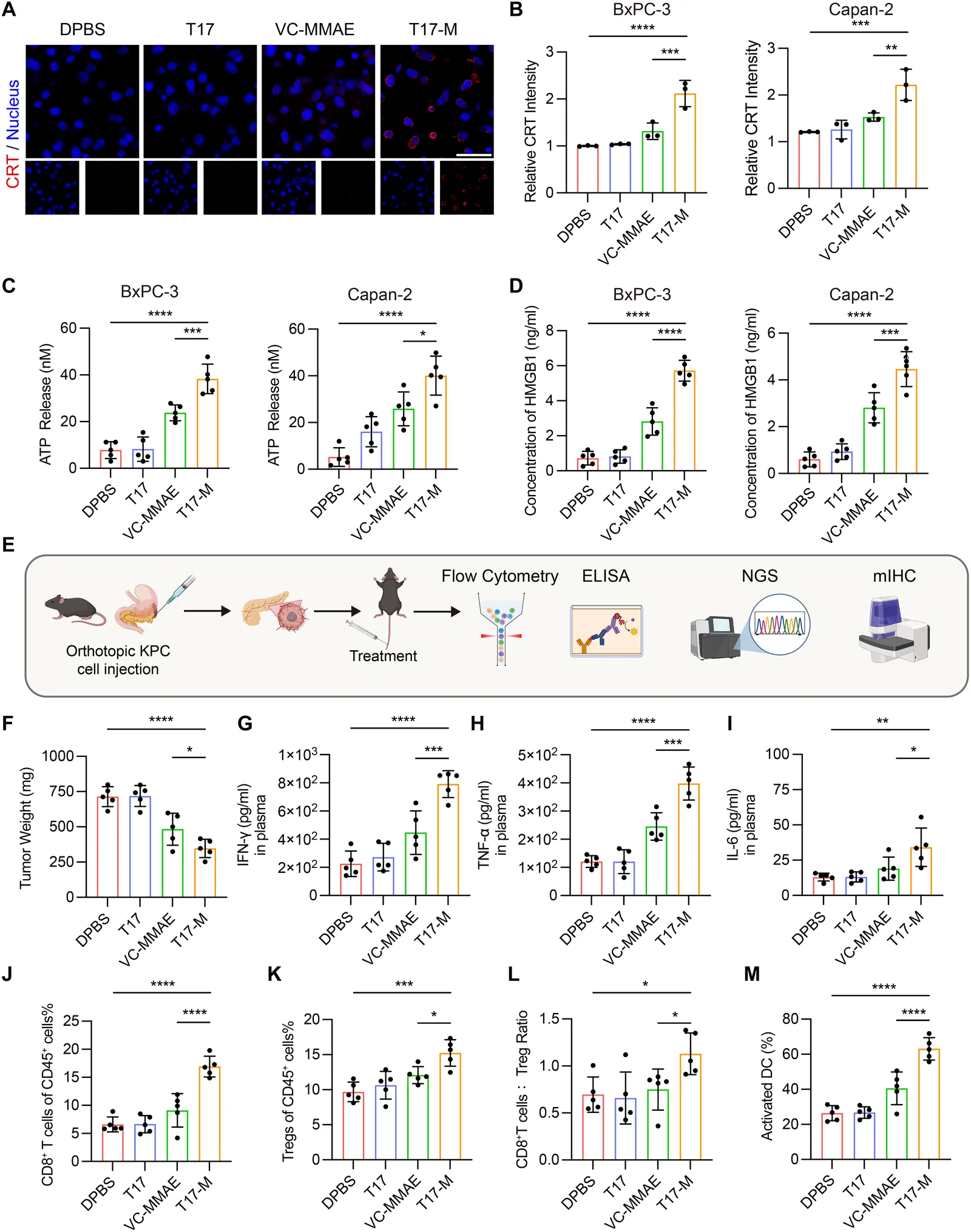
T17-M induces immunogenic cell death and remodels tumor immune microenvironment. (**A**) Confocal images of calreticulin (CRT, red) externalization on BxPC-3 cells after the indicated treatments. Nuclei were stained with Hoechst (blue). Scale bar, 50 μm. (**B**) Quantification of relative CRT intensity on BxPC-3 (left) and Capan-2 (right) cell surfaces detected by flow-cytometry. (**C**) Extracellular ATP release was measured by luciferase-based ATP Assay Kit in BxPC-3 (left) and Capan-2 (right). (**D**) Quantification of HMGB1 concentrations in culture supernatants after indicated treatments. (**E**) Schematic of the orthotopic PDAC model workflow including pancreatic injection of KPC cell, systemic treatment and downstream analyses (flow cytometry, ELISA of plasma cytokines, RNA sequencing, and multiplex IHC). The figure was created using BioRender. 陈, 酯 (2026) https://BioRender.com/tjukooh. (**F**) Final tumor weights of orthotopic model at endpoint for each group. (**G** to **I**) Plasma concentrations of IFN-γ (G), TNF-α (H), and IL-6 (I). (**J** to **M**) Flow cytometric quantification of intratumoral CD8^+^ T cells as a percentage of CD45^+^ cells (J), CD4^+^Foxp3^+^ regulatory T cells as a percentage of CD45^+^ cells (K), the CD8^+^ T cell to regulatory T cell ratio (L), and activated dendritic cells as a percentage of total dendritic cells in tumor-draining lymph nodes (TDLNs) (M). Data are presented as means ± SD (*n* = 3 independent experiments for B, *n *= 5 independent experiments for C and D, and *n *= 5 mice per group for F to M). Statistical significance was assessed using one-way ANOVA followed by Tukey’s multiple-comparisons test. ns, not significant; *P < 0.05, **P < 0.01, ***P < 0.001, and ****P < 0.0001.

To evaluate the antitumor activity of T17-M in an immunocompetent model, we used an established orthotopic mouse model of PDAC. KPC cells, originally derived from a pancreatic tumor arising in a Pdx1^Cre^; Kras^G12D/+^; Trp53^R172H/+^ genetically engineered mouse model (GEMM), were cultured and orthotopically injected into the pancreas of syngeneic C57BL/6J mice. The implanted cells subsequently formed tumors in situ, enabling evaluation of the therapeutic efficacy of T17-M and its effects on the pancreatic tumor immune microenvironment (Fig. 7E) (*47*–*49*). Results show that T17-M markedly reduced endpoint tumor weight (Fig. 7F). Plasma cytokine analysis showed increased IFN-γ and TNF-α concentrations and a more moderate increase in IL-6 after T17-M treatment (Fig. 7, G to I). Flow-cytometric profiling of the tumor immune microenvironment showed that T17-M increased both CD8^+^ T cell and Treg infiltration, with a greater increase in CD8^+^ T cells, resulting in an elevated CD8^+^/Treg ratio (Fig. 7, J to L). Consistent with these findings, multiplex immunohistochemistry further confirmed a marked increase in intratumoral CD8^+^ T cell density following T17-M treatment. Intratumoral CD4^+^ T cell infiltration was also moderately increased compared with that in the DPBS group, although it was not significantly different from that observed following VC-MMAE treatment (fig. S20, A and B). In parallel, the frequency of activated dendritic cells in tumor-draining lymph nodes (TDLNs) was markedly increased (Fig. 7M). Enhanced DC activation may facilitate antigen cross-presentation and the priming of tumor-specific CD8^+^ T cells, consistent with the increased IFN-γ and TNF-α concentrations, enhanced CD8^+^ T cell infiltration, and elevated CD8^+^ T cell/Treg ratio observed after T17-M treatment. Collectively, these data demonstrate that T17-M not only exerts direct cytotoxic activity but also remodels the tumor immune microenvironment.

### T17-M activates antitumor immune programs and enhances the response to PD-1 blockade

Transcriptomic profiling of orthotopic tumors revealed that T17-M induced an ICD-associated inflammatory program. T17-M increased the expression of representative ICD-associated genes, including Panx1, Anxa1, and Hmgb1, while also modulating T cell inhibitory and exhaustion-associated genes, including Ctla4, Lag3, Pdcd1, and Havcr2 (Fig. 8A). Integrated pathway analysis showed increased scores for ICD, antigen presentation, T cell effector activity, and systemic immune responses following T17-M treatment (Fig. 8, B and C). Immune cell deconvolution indicated an increased abundance of activated dendritic cells and a concomitant reduction in immature dendritic cells (Fig. 8D). Consistent with these transcriptomic findings, flow cytometry confirmed an increased frequency of CD80^+^CD86^+^ activated dendritic cells in T17-M–treated tumors (Fig. 8, E and F).

**Fig. 8.**
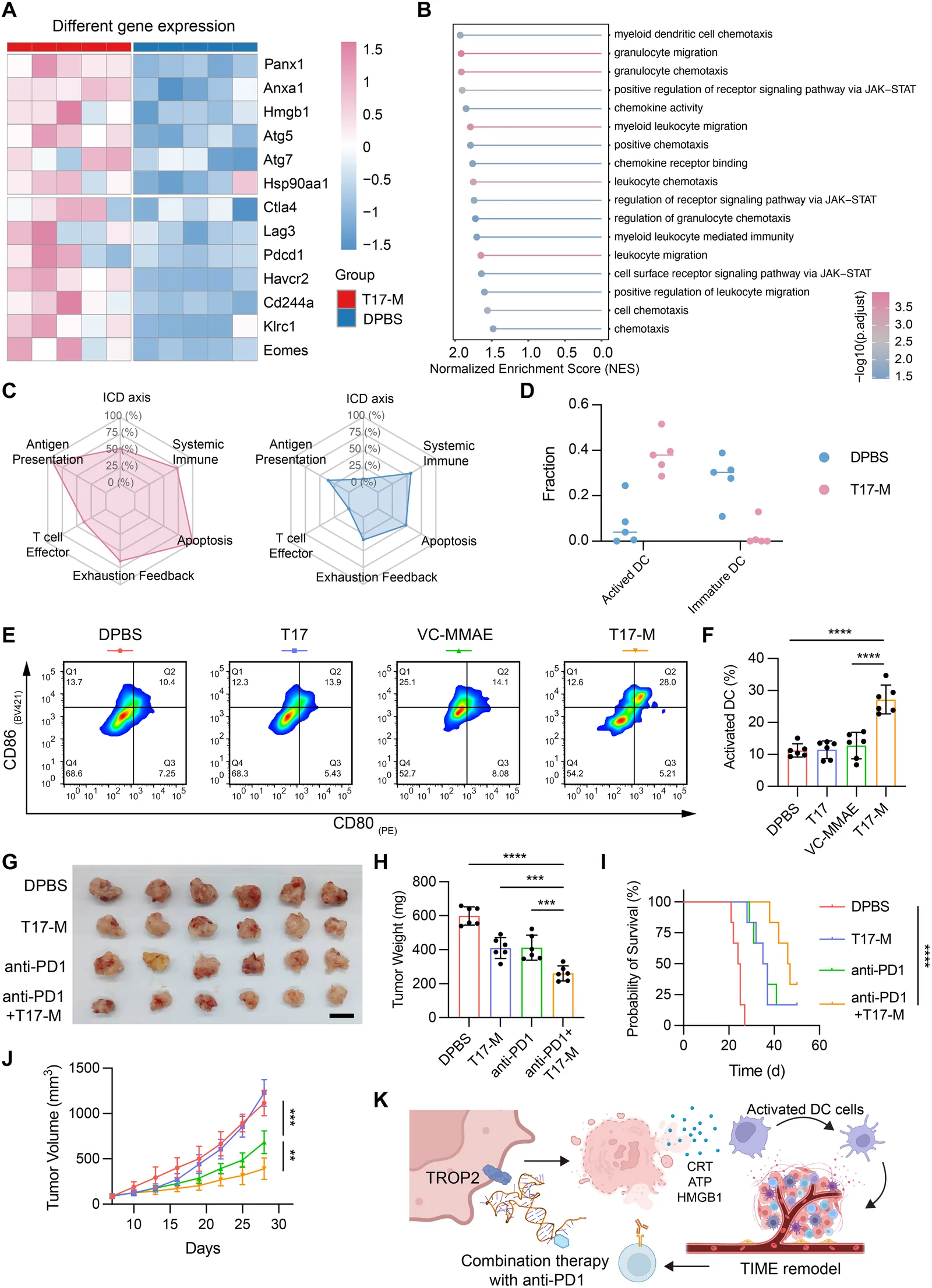
T17-M activates antitumor immune programs and enhances the response to PD-1 blockade. (**A**) Heat map of representative genes related to immunogenic cell death (ICD) and T cell regulation or exhaustion in orthotopic tumors from the T17-M and DPBS groups. (**B**) GSEA lollipop plot contrasting T17-M versus DPBS groups signatures in the orthotopic tumors. (**C**) Percentile-normalized single-sample GSEA (ssGSEA) pathway scores of T17-M (left) and DPBS (right) groups shown as radar plots with percentile normalization. (**D**) Immune-cell deconvolution estimating the fractions of activated and immature dendritic cells in each group. (**E** and **F**) Representative CD80/CD86 flow cytometry plots (E) and quantification of CD80^+^CD86^+^ activated dendritic cells (F) in tumors following the indicated treatments. (**G**) Images of orthotopic tumors from the indicated groups. Scale bar, 1 cm. (**H**) Endpoint tumor weights (*n *= 6). (**I**) Survival curves for the four treatment groups (*n *= 6). (**J**) Tumor growth curve in a subcutaneous model (*n *= 5). (**K**) Schematic of the proposed mechanism: The ApDC-T17-M triggers ICD (CRT exposure, ATP release, HMGB1 secretion), promotes DC activation and antigen presentation, remodels the tumor immune microenvironment (TIME), and cooperates with PD-1 blockade. The figure was created using BioRender. 陈, 酯 (2026) https://BioRender.com/ifbl4cw. Data in (D), (F), (H), and (J) are presented as means ± SD (*n *= 5 tumors per group for A to D, *n *= 6 mice per group for E to I, and *n* = 5 mice per group for J). Statistical significance in (F) and (H) was assessed using one-way ANOVA followed by Tukey’s multiple-comparisons test; selected pairwise comparisons are shown. Tumor volumes at the final experimental time point in (J) were analyzed using one-way ANOVA followed by Tukey’s multiple-comparisons test. Survival differences in (I) were evaluated using the log-rank (Mantel–Cox) test. ns, not significant; *P < 0.05, **P < 0.01, ***P < 0.001, and ****P < 0.0001.

Further immune profiling supported the activation of antigen presenting cell and T cell responses. T17-M increased the frequency of intratumoral XCR1^+^CD11c^+^ conventional type 1 dendritic cells (cDC1s), which are specialized in antigen cross-presentation (fig. S20, C and D). However, the recruited CD8^+^ T cell population also exhibited signs of compensatory dysfunction. T17-M increased the proportion of PD-1^+^TIM-3^+^ exhaustion associated CD8^+^ T cells and was accompanied by reduced proportions of TNF-α^+^IFN-γ^+^ polyfunctional CD8^+^ T cells and decreased granzyme B expression (fig. S20, E to H). Multiplex cytokine profiling of tumor tissues further demonstrated remodeling of the local cytokine milieu following T17-M treatment, including increased signals associated with DC/APC priming and lymphocyte recruitment, together with alterations in T cell effector related and innate/myeloid cytokines (fig. S20I). Collectively, these findings indicate that T17-M induces a locally inflamed tumor immune microenvironment characterized by enhanced immune recruitment and antigen presenting cell activation. Nevertheless, the accompanying increase in exhaustion associated CD8^+^ T cells and reduction in effector function suggest a compensatory dysfunctional response that may restrict sustained antitumor immunity, providing a biological rationale for combination with PD-1 blockade.

We therefore evaluated the therapeutic efficacy of T17-M in combination with anti–PD-1. In the orthotopic KPC model, both T17-M monotherapy and anti–PD-1 treatment reduced tumor burden, whereas the combination produced the greatest reduction in endpoint tumor weight (Fig. 8, G and H). Combination treatment also significantly prolonged survival compared with either monotherapy (Fig. 8I). Consistent antitumor activity was observed in the subcutaneous tumor model, in which the combination produced the strongest suppression of tumor growth (Fig. 8J).

To directly determine the contribution of CD8^+^ T cells to these therapeutic effects, we performed antibody-mediated CD8^+^ T cell depletion in the orthotopic KPC model (fig. S21A). Flow cytometry confirmed efficient CD8^+^ T cell depletion following anti-CD8 treatment (fig. S21B). CD8^+^ T cell depletion significantly attenuated the antitumor efficacy of T17-M and diminished the additional therapeutic benefit of combining T17-M with anti–PD-1 (fig. S21, C and D). Nevertheless, T17-M retained measurable antitumor activity in CD8-depleted mice, indicating that its therapeutic activity comprises both CD8^+^ T cell-dependent and CD8^+^ T cell-independent components. In contrast, the enhanced efficacy of T17-M combined with PD-1 blockade was largely dependent on CD8^+^ T cells. Collectively, these results indicate that T17-M induces direct tumor-cell killing and ICD, promotes dendritic-cell activation and antigen presentation, remodels the tumor immune microenvironment, and enhances the responsiveness of PDAC tumors to PD-1 blockade (Fig. 8K).

## DISCUSSION

In this study, we developed T17-M, a TROP2-targeted aptamer–drug conjugate for selective delivery of MMAE to PDAC cells. T17-M retained the target-binding activity of the parental aptamer, underwent target-associated internalization and lysosomal processing, and showed greater cytotoxic and antitumor activity than the corresponding unconjugated linker–payload control across complementary PDAC models. The mechanism of TROP2-targeted drug delivery by T17-M is illustrated in Fig. 9. Beyond direct tumor-cell killing, T17-M induced hallmarks of immunogenic cell death (ICD), remodeled the tumor immune microenvironment, and enhanced the antitumor activity of PD-1 blockade. These findings provide preclinical proof of concept for T17-M as an alternative TROP2-directed drug-delivery platform in PDAC.

**Fig. 9.**
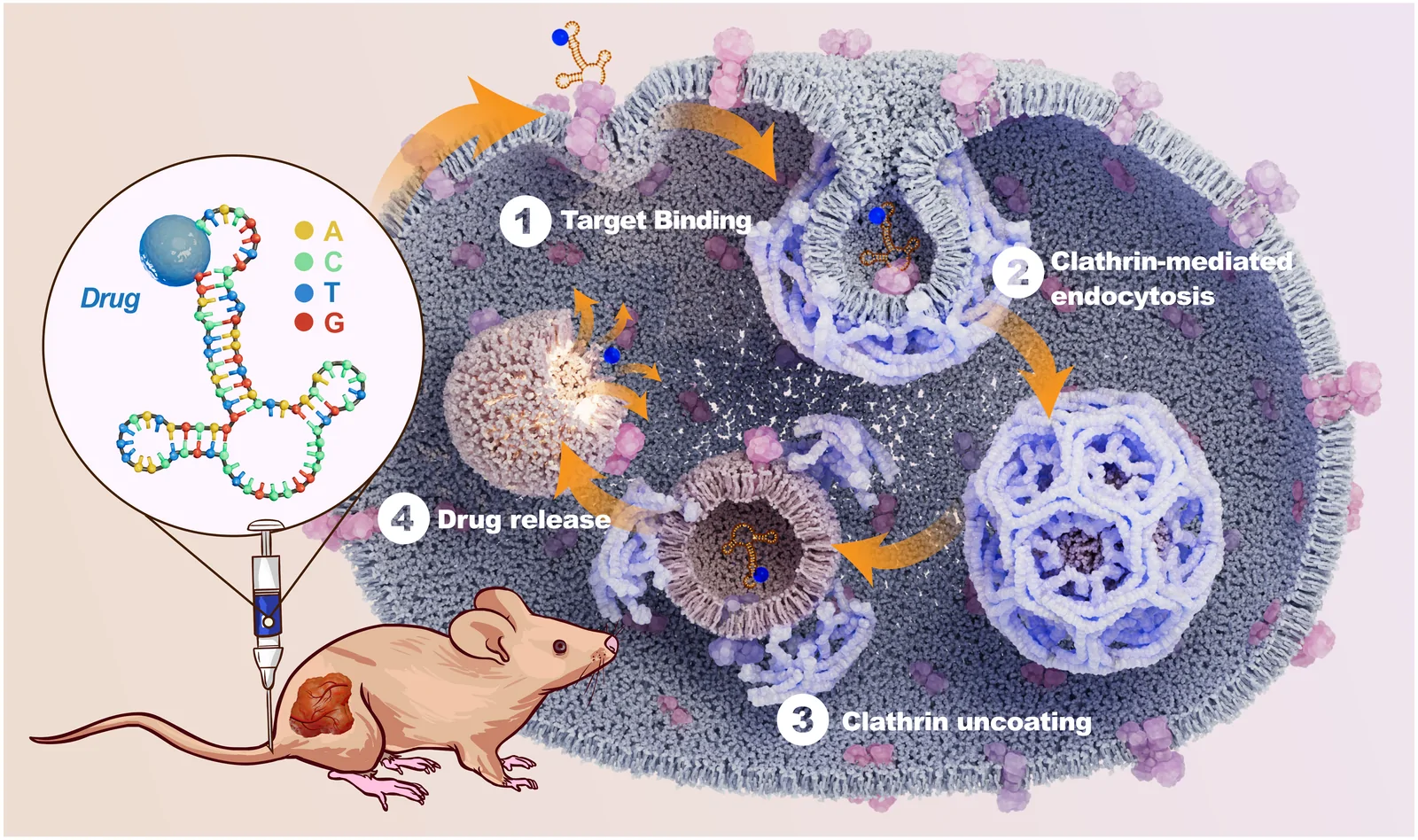
Proposed mechanism of T17-M–mediated targeted drug delivery. T17-M accumulates in tumors and binds membrane TROP2 through the T17 aptamer. Following target-associated internalization through a clathrin-associated endocytic pathway, T17-M traffics to acidic lysosomal compartments, where cathepsin B–dependent linker processing releases the MMAE payload and induces tumor-cell cytotoxicity. The figure was created using Adobe Photoshop (Adobe Inc.).

Membranous TROP2 expression may serve as a candidate biomarker for identifying patients who could potentially benefit from T17-M treatment. In our PDAC cohort, TROP2 expression was detected in 102 of 106 tumor specimens, including strong membranous staining in 44 cases. Higher TROP2 expression was also associated with shorter overall survival, consistent with previous reports linking TROP2 to aggressive biological behavior in pancreatic cancer (*12*). These findings support the prognostic relevance of TROP2 and provide a rationale for the development of TROP2-directed therapies in PDAC. TROP2 depletion reduced the binding of the T17 aptamer, while the cytotoxic advantage of T17-M was also diminished in PDAC cells with relatively low TROP2 expression. Together, these results support the importance of target abundance for effective drug delivery. Therefore, the strong (+++) staining category used in this study should be considered an exploratory definition of TROP2-high disease. Prospective studies are required to establish a clinically applicable assay and an appropriate TROP2 expression threshold for patient selection.

Drug conjugates may also modulate antitumor immunity through treatment-induced cellular stress and ICD. Auristatin-containing ADCs have been shown to induce microtubule disruption, endoplasmic reticulum stress, damage-associated molecular pattern release, dendritic cell activation, and immune-mediated tumor control (*45*, *50*). In our study, T17-M induced calreticulin exposure, extracellular ATP release, and HMGB1 secretion. It also increased activated dendritic cells and intratumoral XCR1^+^CD11c^+^ cDC1s. Both CD8^+^ T cell and Treg infiltration increased following treatment, but the greater increase in CD8^+^ T cells resulted in an elevated CD8^+^ T cell/Treg ratio. Immune activation following T17-M treatment was accompanied by a compensatory dysfunctional CD8^+^ T cell phenotype, characterized by increased PD-1^+^TIM-3^+^ cells, reduced TNF-α^+^IFN-γ^+^ polyfunctional CD8^+^ T cells, and lower granzyme B expression. PD-1 and TIM-3 co-expression has previously been associated with impaired CD8^+^ T cell proliferation and cytokine production (*51*). This dual phenotype provided a biological rationale for combining T17-M with PD-1 blockade. The combination produced greater tumor control and prolonged survival compared with either monotherapy. CD8^+^ T cell depletion attenuated, but did not completely abolish, the antitumor activity of T17-M, indicating the presence of both CD8^+^ T cell–dependent and CD8^+^ T cell–independent components. The residual effect should not be interpreted as completely immune independent because contributions from other immune-cell populations cannot be excluded. In contrast, the additional benefit of PD-1 blockade was largely dependent on CD8^+^ T cells.

A broader trend in oncology is the integration of molecularly targeted therapies with immunotherapy. Beyond directly inhibiting tumor growth, targeted agents can reshape the tumor immune microenvironment by inducing immunogenic cell death, enhancing tumor-antigen release and presentation, activating type I interferon signaling, and promoting effector T cell recruitment. At the same time, treatment-induced immune activation may be accompanied by compensatory up-regulation of immune checkpoints or T cell dysfunction, providing a rationale for combination with immune-checkpoint blockade. This concept is being explored across multiple cancer types, including breast cancer, cervical cancer, Hodgkin lymphoma, and non–small cell lung cancer (*52*–*54*). In the ICARUS-LUNG01 study, response to TROP2-targeted therapy was associated with activation of immune-related pathways and increased intratumoral T cell populations, further illustrating that treatment efficacy may depend on both direct tumor-cell killing and immune remodeling (*55*). Within this framework, the ICD and compensatory CD8^+^ T cell dysfunction observed after T17-M treatment provide a mechanistic basis for combining T17-M with PD-1 blockade in PDAC.

The translational relevance of T17-M was evaluated in cell line–derived xenografts, patient-derived organoids, a PDX model, a liver metastatic-burden model, and a KPC-derived syngeneic orthotopic PDAC model. Pharmacokinetic analysis showed limited circulating exposure to unconjugated MMAE, whereas tissue exposure was highest in the kidney and was also detected in the liver. T17-M was generally tolerated at the therapeutic dose, although elevations in hepatobiliary markers at the highest dose indicate a potential high-exposure safety signal. Rapid systemic clearance has also been observed with other aptamer drug conjugates, highlighting both the reduced prolonged systemic exposure and the potential limitation in tumor exposure associated with this platform (*56*).

Several limitations remain. The exploratory TROP2-high definition requires prospective validation; no matched TROP2-ADC comparison was performed; and the KPC-derived orthotopic model does not fully recapitulate an autochthonous KPC GEMM. In addition, individual cytokine alterations and their cellular sources require further validation, and longer-term toxicology studies are needed. Future work should focus on aptamer stabilization, formulation and dosing optimization, systematic linker–payload screening, and direct comparison with antibody-based TROP2 delivery systems. These findings provide a promising foundation for the further development of T17-M for the treatment of TROP2-positive PDAC and potentially other tumor types with high TROP2 expression.

## MATERIALS AND METHODS

### Cell culture

The human cell lines (BxPC-3, Capan-2, MIA PaCa-2, PANC-1, PATU-8988 T and HPNE) were obtained from ATCC or German Collection of Microorganisms and Cell Cultures (DSMZ). The BxPC-3 and PATU-8988 T were cultured in RPMI 1640, while other cell lines were cultured in DMEM. All of them were cultured in their corresponding media with 10% fetal bovine serum (Procell, catalog no. 164210) and 1% Penicillin Streptomycin (P/S) solution (Yeasen Biotechnology, catalog no. 60162ES76) in a humidified incubator (5% CO_2_, 37°C).

### Gene expression analysis

GEPIA 2.0 (Gene Expression Profiling Interactive Analysis version 2, http://gepia2.cancer-pku.cn/#index) (*23*) was used to assess the expression of TACSTD2 in digestive system tumors and corresponding normal tissue. The bulk RNA-sequencing data of 182 pancreatic adenocarcinoma samples were collected from The Cancer Genome Atlas (TCGA). Samples were assigned into high and low TACSTD2 expression groups according to the median. Differentially expressed genes were calculated through the R package “DESeq2”. Gene Set Enrichment Analysis (GSEA) was conducted using the R package “clusterProfiler” (*57*) by setting the 50 canonical cancer hallmarks as reference (*58*).

### Single-cell RNA sequencing analysis

The single-cell RNA sequencing data of 24 PDAC samples and 11 adjacent normal tissues and corresponding cell type annotation files were obtained from Genome Sequence Archive with the accession number CRA001160 (*24*). Putative doublets were detected and removed using the R package “Doublet Finder” (*59*). Quality control criteria were as follows: 1) cells had either fewer than 100 or greater than 8,000 RNA features, 2) less than 500 RNA counts, or 3) over 10% RNA features derived from the mitochondrial genome were removed. Filtered gene expression profiles were read, normalized with the “Log Normalized” method, scaled by regressing out the cell-cycle signature scores, and subsequently integrated to minimize the batch effect with the R package “harmony” after principal component analysis (*60*). The top 10 dimensions from harmony were selected to perform UMAP dimensionality reduction. Visualization was conducted through the R packages “Seurat” and “ggplot2”.

### Immunohistochemical (IHC) staining and HE staining

All samples were assessed by two independent pathologists. IHC staining was performed to evaluate TROP2 expression in PDAC tissues, which were pathologically confirmed by the Department of Pathology, Renji Hospital (Shanghai, China) between August 2020 and February 2022. The use of human FFPE tissue samples and associated clinical information was approved by the Ethics Committee of Renji Hospital, Shanghai Jiao Tong University School of Medicine (approval number: KY2024–065-C). Informed consent was obtained from all patients for the research use of their tissue specimens. The formalin-fixed paraffin-embedded (FFPE) tissues were cut at 4 μm thickness, and the staining was accomplished by Leica Bond-RXm staining platforms. The scoring system we used was previously published (*61*, *62*). Briefly, the staining intensity was evaluated as follows: 0, negative; 1, low; 2, medium; 3, high. The staining extent was evaluated as follows: 0, 0%; 1, 1–25%; 2, 26–50%; 3, 51–100%. The final score was represented by the staining intensity multiplied by staining extent, and was divided into four levels: 0, negative (−); 1–2, low staining (+); 3–5, medium staining (++); 6–9, high staining (+++). For the HE stain, the collected tissues fixed in 10% formalin were dehydrated by Leica ASP 300 S Tissue Processor, and the HE staining was finished by Leica ST5010 Autostainer XL. All sections were observed using Leica DM6 B Upright Microscopes.

### SELEX procedure

The 76 nt ssDNA library containing a 32 nt random sequence domain (5’-CTACGGTGCCTTGAAGTGACTCG- 32 nt random nucleotide -TCATAGCAGGTCACTTCCAGG-3′) was used as the initial ssDNA pool for subsequent aptamer screening. At first, the TROP2 protein was fastened to magnetic beads, the beads were washed once with washing buffer. TROP2 beads were used as a positive target and the blank beads were used as a negative target. During the first round of selection, 500 pM ssDNA library dissolved in 280 μl binding buffer (containing 5 mM MgCl_2_ and 0.05 mg/ml hsDNA), annealed at 80°C, and gradually cooled to room temperature to allow structural folding. Then, the library was mixed with 50 μl TROP2 beads and incubated for 1 h. A magnetic frame was used to restore the beads and the supernatant which containing unbound DNA was removed. Then, the TROP2 beads were separated, and the last ssDNA pool were incubated with negative target, and the supernatant were collected as templates for PCR amplification. After amplification the obtained ssDNA library was entered into next round selection. To improve the affinity and specificity, the SELEX procedure was given following selection pressure: 1) The incubation time with positive targets was gradually shortened, meanwhile the incubation time with negative targets was gradually prolonged; 2) The concentration of positive target was gradually decreased; 3) During the incubation of round 5 and round 7 we added 5% FBS to improve the biological similarity with blood circulation.

### Computational modeling study

The secondary structures and the tertiary structures of TROP2–1 and TROP2–17 were built in RNAfold webserver and RNAComposer. For molecular dynamics simulations (MDS), the structure of TROP2 was downloaded from Protein Data Bank (PDB). The analysis procedure had been reported previously (*63*).

### Synthesis and characterization of T17-M

T17 and its derivatives were synthesized and purified by Sangon Biotech Co., Ltd. (Shanghai, China). T17-M was prepared by conjugating thiol-functionalized T17 with maleimide-functionalized MC–Val–Cit–PAB–MMAE through thiol–maleimide coupling chemistry. Briefly, thiol-functionalized T17 was dissolved in DPBS containing 1 mM tris(2-carboxyethyl) phosphine (TCEP) and incubated for 1 h to reduce disulfide bonds and generate free thiol groups. After removal of excess TCEP, maleimide-functionalized MC–Val–Cit–PAB–MMAE, dissolved in acetonitrile, was added at a fivefold molar excess relative to the aptamer. The reaction mixture was incubated at room temperature overnight with gentle agitation. The resulting T17–MMAE conjugate (T17-M) was purified by high-performance liquid chromatography and characterized by mass spectrometry and analytical high-performance liquid chromatography.

### IC_50_ assay and long-term cell proliferation assay

For IC_50_ assay, PDAC cells were seeded in 96-well plates (4000 cells/well) and allowed to adhere for 24 h. The next day, cells were incubated with gradient concentration of VC-MMAE or T17-M for 72 h. After that, the cell viability was analyzed by Cell Counting Kit-8 (CCK8, Vazyme, catalog no. A311–01) assay, the 450 nm absorbance was measured by Synergy 2 (BioTek, Winooski, Vermont, USA). For long-term cell proliferation assay, cells were seeded in a 12-well at a density of 2 × 10^4^ per well and were incubated in medium containing variable concentrations of indicated treatments for 10–14 days. After that, the colonies were fixed with 4% paraformaldehyde and stained with crystal violet (Beyotime, catalog no. C0121).

### Western blotting assay

The cells were washed by PBS and lysed by RIPA lysis buffer (Beyotime, catalog no. P0013B) mixed with protease cocktail inhibitor (Yeasen Biotechnology, catalog no. 20109ES05). The concentration of cell lysates was determined by BCA Protein Assay Kit (Beyotime, catalog no. P0009). After that, 10 μg protein was separated by SDS-PAGE and transferred to PVDF membranes. The membranes were blocked by 5% skimmed milk and incubated with primary antibody [TROP2 Rabbit mAb (CST, catalog no. 47866) and GAPDH Rabbit mAb (ABclonal, catalog no. A19056)] at 4°C overnight, then the goat anti-rabbit secondary antibody was added at room temperature after three washes by TBST. At last, the chemiluminescent signals of the protein on membranes were detected by ChemiDoc Imaging System.

### Immunofluorescence staining

Cells were seeded on cover slips in 6-well plate with complete medium and allowed to adhere for 24 h. After rinsed by PBS three times, cells were fixed by 4% paraformaldehyde at room temperature for 15 min. The specimens were incubated in primary antibody overnight at 4°C after blocked in Blocking Buffer, which contained 5% normal goat serum and 0.1% Triton X-100, for 60 min. Afterward we incubated specimens in fluorochrome-conjugated secondary antibody (CST, catalog no. 4412) and sealed the cover slips with Antifade Reagent with DAPI (Shanghai Life-iLab Biotech, catalog no. AC28L533). The images were taken by the confocal microscopy (TCS SP8, Leica).

### Dissociation constant (Kd) measurement assay and flow cytometry analysis

For the Kd measurement, the Aldehyde/Sulfate latex microspheres (Thermo Fisher Scientific, catalog no. A37478) were incubated with Recombinant Human TROP2 (ABclonal, catalog no. RP02570) overnight at 4°C. After centrifugation (6000 rpm, 3 min), the microspheres were blocked by the blocking buffer A (DPBS with 20% BSA and 100 μM Glycine) for 1 hour and blocking buffer B [DPBS with 5% BSA and 1% tRNA (Solarbio, catalog no. T8630)] for another hour. Then, the microspheres were incubated with the aptamers in gradient concentration for 1 hour at 4°C. At last, the fluorescence intensity of microspheres was detected by flow cytometry.

### siRNA-mediated TROP2 gene silencing assay

The siRNA of TROP2 (siRNA-1: 5’AUAAACGUCUCCUUUGCGCCG-3′ and siRNA-2: UACAAGCUCGGUUCCUUUCUC-3′) and si-NC were purchased from Genomeditech (Shanghai, China). Cells were transfected with siRNA by Rfect transfection reagent (BAIDAI) following the transfection protocol.

### Apoptosis assay

The cells were seeded in a 6-well plate and received treatment of T17, VC-MMAE, and T17-M (100 nM) for 24 hours. Then, the annexin V-FITC Apoptosis Detection Kit (Beyotime, catalog no. C1062S) was used to explore the apoptosis of cells. The stained cells were detected by flow cytometry.

### In vivo animal assay

All animal experiments were approved by the Institutional Animal Care and Use Committee (IACUC) of Renji Hospital, Shanghai Jiao Tong University School of Medicine (approval number: RJ-2024-149B). To establish two cell-derived xenograft models, 3 × 10^6^ BxPC-3 or Capan-2 cells were resuspended in 100 μl of DPBS and injected subcutaneously into female BALB/c nude mice aged 4 to 6 weeks. For the ex vivo imaging, Cy5 labeled aptamers or aptamer drug conjugate were administered by intravenous injection for three mice per group. At certain time points, we obtained tumors and major organs, and the fluorescence images were taken by IVIS Lumina III in vivo imaging system (PerkinElmer, USA). For the anti-tumor activity evaluation, CDXs received treatments 12 days after implantation, when the tumor volume approached 80 mm3. Mice were divided into four groups randomly (DPBS, T17, VC-MMAE, and T17-M). The mice were injected through tail vein with DPBS or 250 nmol/kg of T17, VC-MMAE, and T17-M. The tumor volumes were calculated using the equation: volume (mm^3^) = length × width^2^/2. For the safety evaluation, the ICR mice (female, 6–8 weeks old) received 1000 nmol/kg of T17-M, which was four times the therapeutic dosage, and the index of complete blood count (CBC), liver function and kidney function were measured at 1 day, 14 days or 28 days after injection. For the patient-derived xenograft (PDX) model, fresh pancreatic ductal adenocarcinoma specimens obtained during surgical resection were subcutaneously implanted into immunodeficient mice to establish patient-derived xenograft tumors. The tumors were subsequently expanded by serial transplantation, and early-passage PDX tumor fragments were used for therapeutic evaluation. For biodistribution analysis, mice bearing PDX tumors received intravenous injections of Cy5-labeled T17 or T17-M, and fluorescence images were acquired before injection and at 1, 2, and 3 h after injection using an IVIS Lumina III imaging system. Tumors and major organs were subsequently collected for ex vivo fluorescence imaging. For therapeutic studies, PDX tumor fragments were subcutaneously implanted into BALB/c nude mice. When tumor volumes reached approximately 80 mm^3^, mice were randomly assigned to four groups (*n *= 6 per group) and intravenously treated with DPBS or T17, VC-MMAE, or T17-M at 250 nmol/kg on days 15, 18, 21, and 24 after tumor implantation. Tumor dimensions were measured using calipers, and tumor volumes were calculated as length × width^2^/2. Mice were euthanized on day 36, and tumors were collected for tumor weight measurement and histological and immunohistochemical analyses.

### In vivo fluorescence imaging and biodistribution

Cy5-labeled aptamers or conjugates were administered intravenously to tumor-bearing mice. Whole-body fluorescence was recorded using an IVIS Lumina III imaging system (PerkinElmer) under identical acquisition settings. For comparison of TROP2–1 and TROP2–17, mice were euthanized 2 h after injection, and tumors and major organs were imaged ex vivo. Average radiant efficiency was quantified using Living Image software after background subtraction.

### Repeated-dose safety assessment in Sprague–Dawley rats

Sprague–Dawley rats were randomized to saline or low-, middle-, or high-dose T17-M groups (*n *= 3 per group). T17-M was administered intravenously on days 1, 5, 9, and 13 (q4d × 4) at low (250 nmol/kg), middle (750 nmol/kg), and high doses (1250 nmol/kg). Body weight was monitored throughout the study. Blood was collected on day 15 for hematologic analysis, including WBC, PLT, neutrophils, lymphocytes, monocytes, RBC, hemoglobin, MCV, MCH, and MCHC, and for serum chemistry, including ALT, AST, ALP, γ-glutamyl transferase, total bilirubin, direct bilirubin, total bile acids, albumin, urea, and creatinine. Rats were euthanized after blood collection, and the heart, lung, liver, spleen, and kidney were collected for H&E examination.

### Pharmacokinetic and tissue distribution analysis

Sprague–Dawley rats received a single intravenous administration of T17-M, and anticoagulated blood samples were collected at 0.5, 1, 2, 4, 8, 16, and 24 h after administration. Plasma was separated by centrifugation, and total and free MMAE concentrations were quantified by ultraperformance liquid chromatography–tandem mass spectrometry (UPLC–MS/MS). For tissue distribution analysis, the heart, liver, spleen, lungs, and kidneys were collected at the indicated time points, weighed, homogenized, and subjected to protein precipitation before analysis. MMAE concentrations were calculated using an external standard curve and expressed as ng/ml plasma or ng/g tissue.

### KPC-derived syngeneic orthotopic PDAC model

KPC cells, originally derived from a pancreatic tumor arising in a Pdx1^Cre^; Kras^G12D/+^; Trp53^R172H/+^ mouse (*47*), were resuspended in 1:1 medium/Matrigel mixture (BD Biosciences, catalog no. 354248). A total of 2 × 10^5^ cells in 50 μl were injected into the pancreatic tail of anesthetized syngeneic C57BL/6J mice through a small left abdominal incision. The pancreas was returned to the abdominal cavity, and the incision was closed in layers. For immune-profiling experiments, mice received DPBS, T17, VC–MMAE, or T17-M at 250 nmol/kg intravenously, and tumors, plasma, tumor-draining lymph nodes, and spleens were collected at endpoint.

### RNA-seq and analysis

Total RNA was extracted from tissue samples using a magnetic bead–based RNA extraction kit (Servicebio, catalog no. G3690). RNA purity was assessed using a NanoDrop spectrophotometer, RNA concentration was quantified using the Equalbit RNA BR Assay Kit (Vazyme, catalog no. EQ212–02), and RNA integrity was evaluated by agarose gel electrophoresis. Approximately 2 μg of total RNA from each sample was used for library preparation. Messenger RNA was enriched using the Hieff NGS mRNA Isolation Master Kit V2 (Yeasen Biotechnology, catalog no. 12629ES96), and sequencing libraries were constructed using the Hieff NGS EvoMax RNA Library Prep Kit (Yeasen Biotechnology, catalog no. 12341ES97) according to the manufacturer’s instructions. Libraries were circularized using the Hieff NGS Dual Barcode Fast-Pace DNA Cyclization Kit for MGI (Yeasen Biotechnology, catalog no. 13340ES96) and sequenced on a DNBSEQ-T7 platform (MGI Tech) to generate 150-bp paired-end reads. Raw reads were processed using fastp for adapter removal, quality control, and filtering of low-quality reads. Clean reads were aligned to the appropriate reference genome using HISAT2 (version 2.2.1), and gene-level read counts and transcripts per kilobase million values were generated using StringTie (version 2.2.1). Differential expression analysis was performed on raw gene-count data using the DESeq2 R package (version 1.42.1), and genes with an absolute log2 fold change greater than 1 and a Benjamini–Hochberg–adjusted *P* value less than 0.01 were considered significantly differentially expressed. Gene Ontology and Kyoto Encyclopedia of Genes and Genomes pathway enrichment analyses were performed using the clusterProfiler R package (version 4.10.1), with an adjusted *P* value less than 0.05 considered statistically significant. Gene set enrichment analysis was additionally performed using clusterProfiler to identify biological pathways and functional gene sets associated with the transcriptional differences between groups.

### Preparation of single-cell suspensions and flow cytometry

Fresh tumors were minced and digested in medium containing collagenase IV and DNase I at 37°C for 35 min with agitation. Digested tissues were passed through 70-μm cell strainers, washed, and subjected to red blood cell lysis when necessary. Tumor-draining lymph nodes (TDLNs) and spleens were mechanically dissociated and passed through 70-μm cell strainers to obtain single-cell suspensions. For intracellular cytokine analysis, cells were stimulated ex vivo with phorbol 12-myristate 13-acetate (PMA) for 4 h in the presence of a protein transport inhibitor. Cells were subsequently stained with BD Horizon Fixable Viability Stain 780 (FVS780; BD Biosciences, catalog no. 565388) and incubated with an anti-mouse CD16/CD32 Fc receptor–blocking antibody before surface staining. Following surface staining, cells were fixed and permeabilized and then stained intracellularly for IFN-γ, TNF-α, and granzyme B. Foxp3 staining was performed in a separate panel using a transcription factor fixation and permeabilization protocol. The antibodies used across the flow cytometry experiments included BV650 anti-mouse CD45 (BD Biosciences, catalog no. 563410); BV510 anti-mouse CD3ε (BD Biosciences, catalog no. 563024); PE-Cy7 and BV605 anti-mouse CD4 (BD Biosciences, catalog nos. 552775 and 563151, respectively); FITC and RY703 anti-mouse CD8a (BD Biosciences, catalog nos. 553030 and 571447, respectively); PE anti-mouse CD25 (BD Biosciences, catalog no. 553075); Alexa Fluor 647 anti-mouse Foxp3 (BD Biosciences, catalog no. 560401); APC and BB700 anti-mouse PD-1 (BD Biosciences, catalog nos. 562671 and 566514, respectively); PE and BV421 anti-mouse TIM-3 (BD Biosciences, catalog nos. 568428 and 747626, respectively); BV421 anti-mouse IFN-γ (BD Biosciences, catalog no. 563376); BB700 anti-mouse TNF-α (BD Biosciences, catalog no. 566510); PE anti-mouse granzyme B (Thermo Fisher Scientific, catalog no. 12–8898-82); FITC anti-mouse CD11b (BD Biosciences, catalog no. 557396); FITC and BV480 anti-mouse CD11c (BD Biosciences, catalog nos. 557400 and 565627, respectively); BB700 anti-mouse I-A/I-E (BD Biosciences, catalog no. 746197); PE anti-mouse CD80 (BD Biosciences, catalog no. 553769); PE-Cy7 and BV421 anti-mouse CD86 (BD Biosciences, catalog nos. 560582 and 564198, respectively); BV605 anti-mouse CD40 (BD Biosciences, catalog no. 745218); and BV510 anti-mouse/rat XCR1 (BioLegend, catalog no. 148218). Regulatory T cells were defined as live CD45^+^CD3^+^CD4^+^Foxp3^+^ cells. Conventional type 1 dendritic cells (cDC1s) were defined as live CD45^+^CD11c^+^MHC-II^+^XCR1^+^ cells, and activated dendritic cells were defined as live CD45^+^CD11c^+^MHC-II^+^CD80^+^CD86^+^ cells. CD8^+^ T cells with an exhaustion-associated phenotype were defined as live CD45^+^CD3^+^CD8^+^PD-1^+^TIM-3^+^ cells. Flow cytometric data were acquired using a BD LSRFortessa X-20 flow cytometer and analyzed with FlowJo software.

### Multiplex immunofluorescence staining

Paraffin-embedded tumor sections from the indicated treatment groups were subjected to sequential immunofluorescence staining using a tyramide signal amplification method. Sections were deparaffinized, rehydrated through graded ethanol, and subjected to heat-induced antigen retrieval in EDTA buffer (pH 9.0) under high-pressure conditions. Endogenous peroxidase activity was blocked with 3% hydrogen peroxide for 10 min at room temperature, and nonspecific binding was blocked with 10% normal goat serum for 30 min at 37°C. Sections were first incubated with rabbit anti-CD4 antibody (Abcam, catalog no. ab183685; 1:4000) overnight at 4°C, followed by goat anti-rabbit IgG H&L conjugated to horseradish peroxidase (Abcam, catalog no. ab205718; 1:4000) for 45 min at 37°C. CD4 signals were developed using iFluor 488 tyramide (AAT Bioquest, catalog no. 11060; 1:600) for 10 min at room temperature. Bound antibodies were subsequently removed by microwave treatment in citrate antigen-retrieval buffer for 5 min. Sections were then incubated with rabbit anti-CD8 antibody (Abcam, catalog no. ab209775; 1:4000) overnight at 4°C, followed by the same horseradish peroxidase–conjugated secondary antibody and Cy5 tyramide (AAT Bioquest, catalog no. 11066; 1:600). Nuclei were counterstained with DAPI (Solarbio, catalog no. C0060; 1:500) for 5 min, and sections were mounted with fluorescence mounting medium. Fluorescence images were acquired using an Olympus BX53 fluorescence microscope and a Pannoramic MIDI imaging system. CD4, CD8, and nuclear signals were visualized in green, red, and blue, respectively.

### Multiplex cytokine and chemokine profiling of tumor lysates

Freshly isolated tumors were weighed and homogenized on ice in prechilled lysis buffer at a ratio of 200 to 300 μl per 10 mg of tissue. The lysis buffer contained 150 mM NaCl, 20 mM Tris-HCl, 1 mM EDTA, 1 mM EGTA, and 1% Triton X-100 (pH 7.5) and was supplemented with protease inhibitors. Tissue homogenates were incubated at 4°C for 20 min and centrifuged at 13,000 rpm for 10 min at 4°C. The clarified supernatants were collected, and total protein concentrations were determined using a bicinchoninic acid assay. Tumor lysates were stored at −80°C until analysis. Cytokine and chemokine concentrations in tumor lysates were measured using the Bio-Plex Pro Mouse Cytokine 23-Plex Assay (Bio-Rad Laboratories, M60-009RDPD) according to the manufacturer’s instructions. The panel simultaneously quantified IL-1α, IL-1β, IL-2, IL-3, IL-4, IL-5, IL-6, IL-9, IL-10, IL-12p40, IL-12p70, IL-13, IL-17A, eotaxin/CCL11, granulocyte colony-stimulating factor (G-CSF), granulocyte-macrophage colony-stimulating factor (GM-CSF), interferon-γ (IFN-γ), keratinocyte-derived chemokine (KC/CXCL1), monocyte chemoattractant protein-1 (MCP-1/CCL2), macrophage inflammatory protein-1α (MIP-1α/CCL3), MIP-1β/CCL4, regulated on activation, normal T cell expressed and secreted (RANTES/CCL5), and tumor necrosis factor-α (TNF-α). Tumor lysates and serially diluted standards were incubated with analyte-specific antibody-coupled magnetic beads. After washing, the beads were sequentially incubated with biotinylated detection antibodies and streptavidin–phycoerythrin. Following a final washing step, the beads were resuspended in sheath fluid, and fluorescence signals were acquired using a Luminex 200 system. Analyte concentrations were calculated from the corresponding standard curves using MILLIPLEX Analyst software (version 5.1), normalized to the total protein content of each tumor lysate, and expressed as picograms per milligram of total protein. Assay runs were accepted when the coefficient of determination of the standard curve was greater than 0.99, the recovery of standards within the linear range was between 70 and 130%, and the coefficient of variation of standard replicates within the linear range did not exceed 25%. For heatmap visualization, normalized analyte concentrations were log_10_ (x + 1)–transformed and subsequently standardized for each analyte across all samples using row-wise Z-score normalization.

### Statistical analysis

Statistical analyses were performed using GraphPad Prism version 9.0 (GraphPad Software, USA). Comparisons between two independent groups were performed using an unpaired, two-tailed Student’s *t* test. Comparisons among multiple groups were performed using one-way or two-way analysis of variance (ANOVA), followed by the appropriate multiple-comparisons test, as specified in the corresponding figure legends. Dose–response curves were fitted by nonlinear regression using a four-parameter logistic model, and survival curves were compared using the log-rank (Mantel–Cox) test. Data are presented as means ± SD unless otherwise stated, and all statistical tests were two-sided. Significance levels: ns, not significant; *P < 0.05, **P < 0.01, ***P < 0.001, and ****P < 0.0001.
