# Supplementary material for: A TROP2-targeting aptamer enables selective drug delivery and potent antitumor activity in pancreatic cancer
Source: Sci Adv. 2026 Nov 11;12(46):eaee3585. doi: 10.1126/sciadv.aee3585 (PMC13754016; doi:10.1126/sciadv.aee3585)
Supplement: Supplementary file 1 [file sciadv.aee3585_sm.pdf]

Science Advances
AAAS

Supplementary Materials for

A TROP2-targeting aptamer enables selective drug delivery and potent antitumor activity in pancreatic cancer

Yuanding Liu *et al.*

Corresponding author: Yingbin Liu, laoniulyb@shsmu.edu.cn; Yang Sun, suny1989@sjtu.edu.cn; Weihong Tan, tan@hnu.edu.cn

Sci. Adv. **12**, eaee3585 (2026)
DOI: 10.1126/sciadv.aee3585

This PDF file includes:

Figs. S1 to S21
Tables S1 and S2

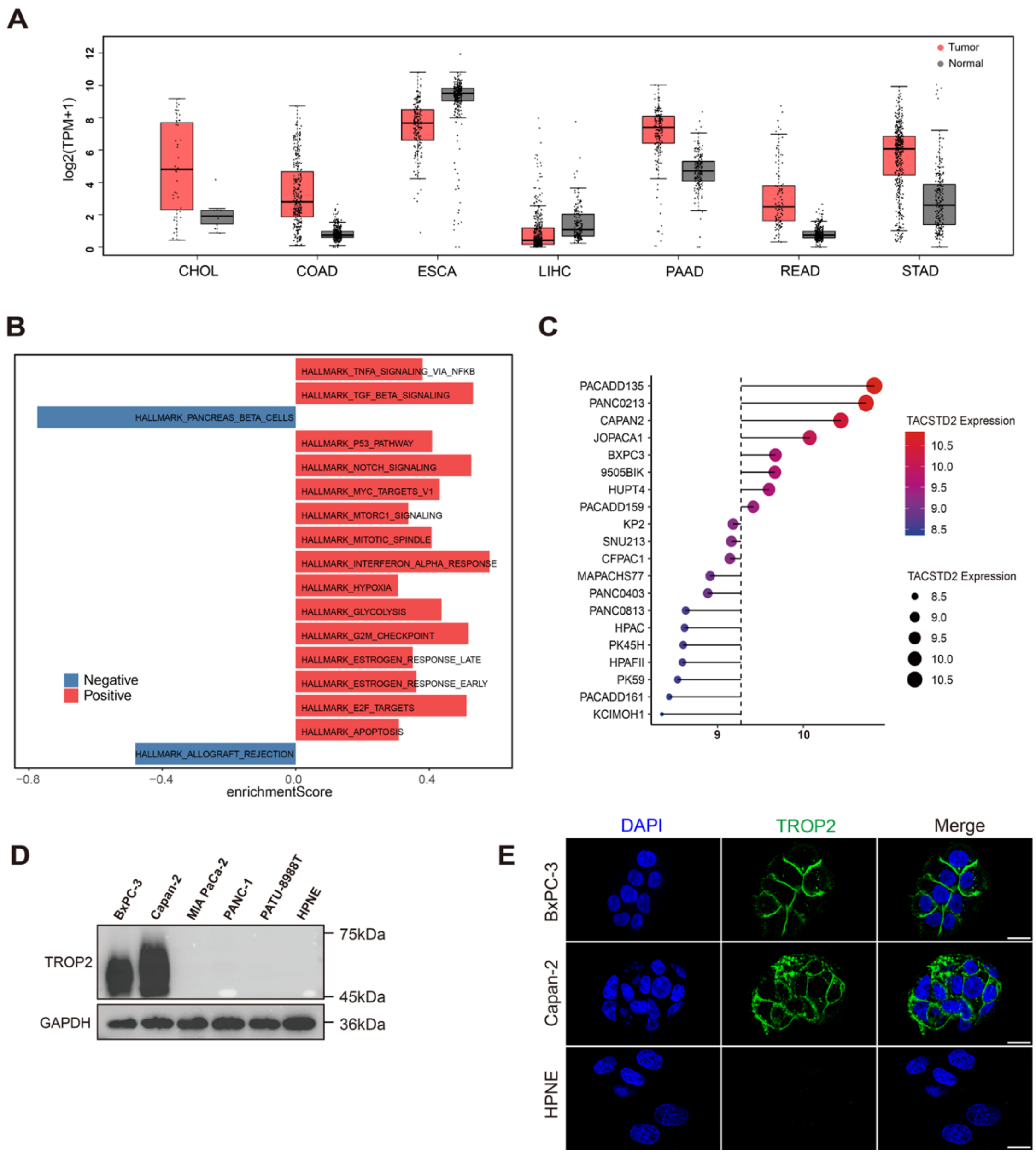

Fig. S1. Transcriptomic and protein-expression profiling of TROP2 (TACSTD2) in PDAC. (A) The transcriptional expression level of TACSTD2 in digestive system tumors and their corresponding normal tissue. Analyzed by online database GEPIA 2.0 (http://gepia2.cancer-pku.cn/#index) **(B)** The Gene Set Enrichment Analysis (GSEA) of PDAC which possessed higher TACSTD2 expression level. **(C)** Top 20 PDAC cell lines which have high TACSTD2 expression level according to CCLE dataset. **(D)** The TROP2 protein level in common PDAC cell lines and normal pancreatic ductal epithelial cell line HPNE assessed by western blotting assay. **(E)** Representative immunofluorescence staining images of TROP2 (green) in cell lines. Scale bar: 20μm.

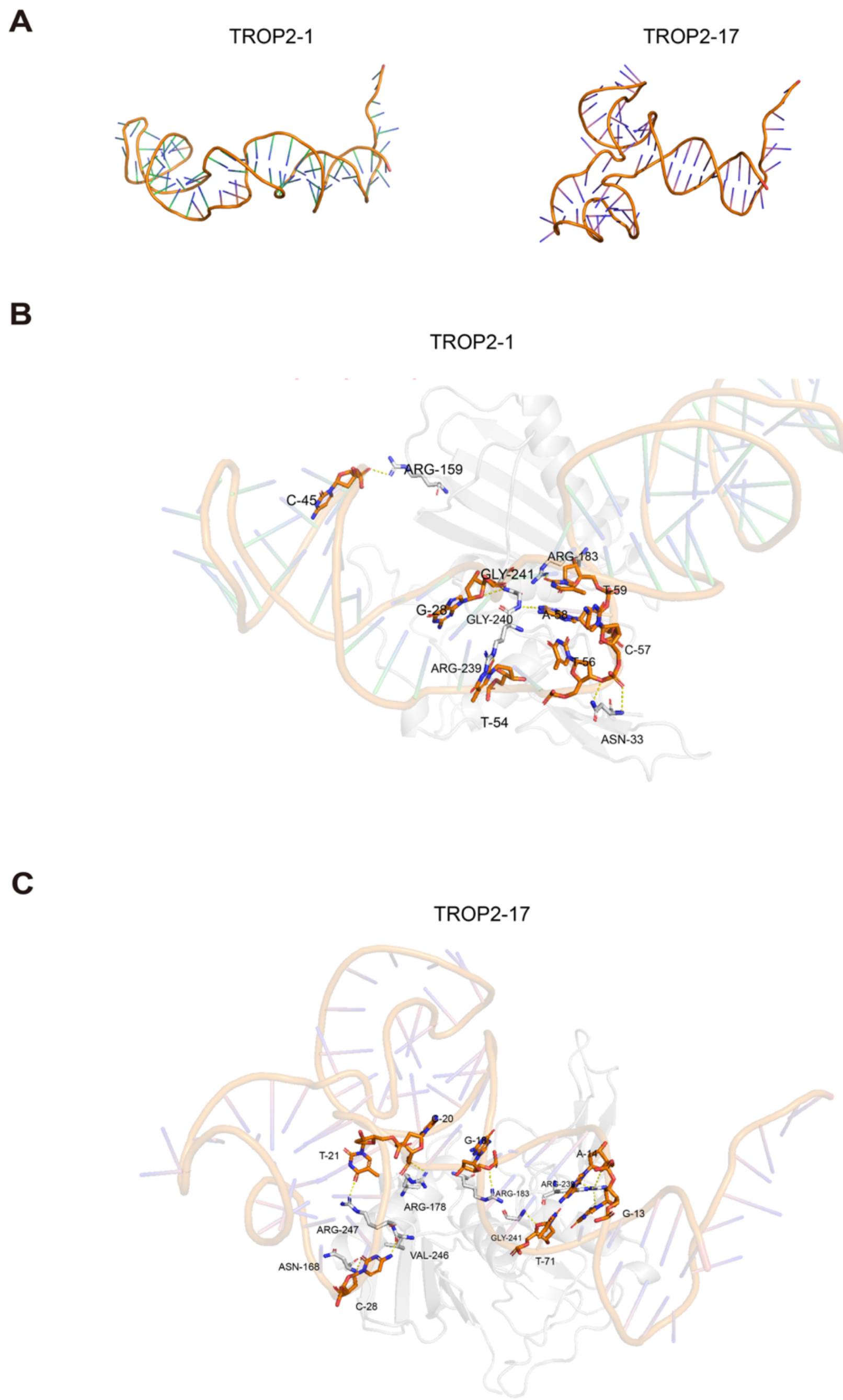

Fig. S2. Predicted Structural models of TROP2 aptamers and their binding to the TROP2 extracellular TROP2 domain. (A) 3D tertiary structures of TROP2-1 and TROP2-17. **(B, C)** The detail of the binding between TROR2-1 or TROP2-17 and TROP2 extracellular domain.

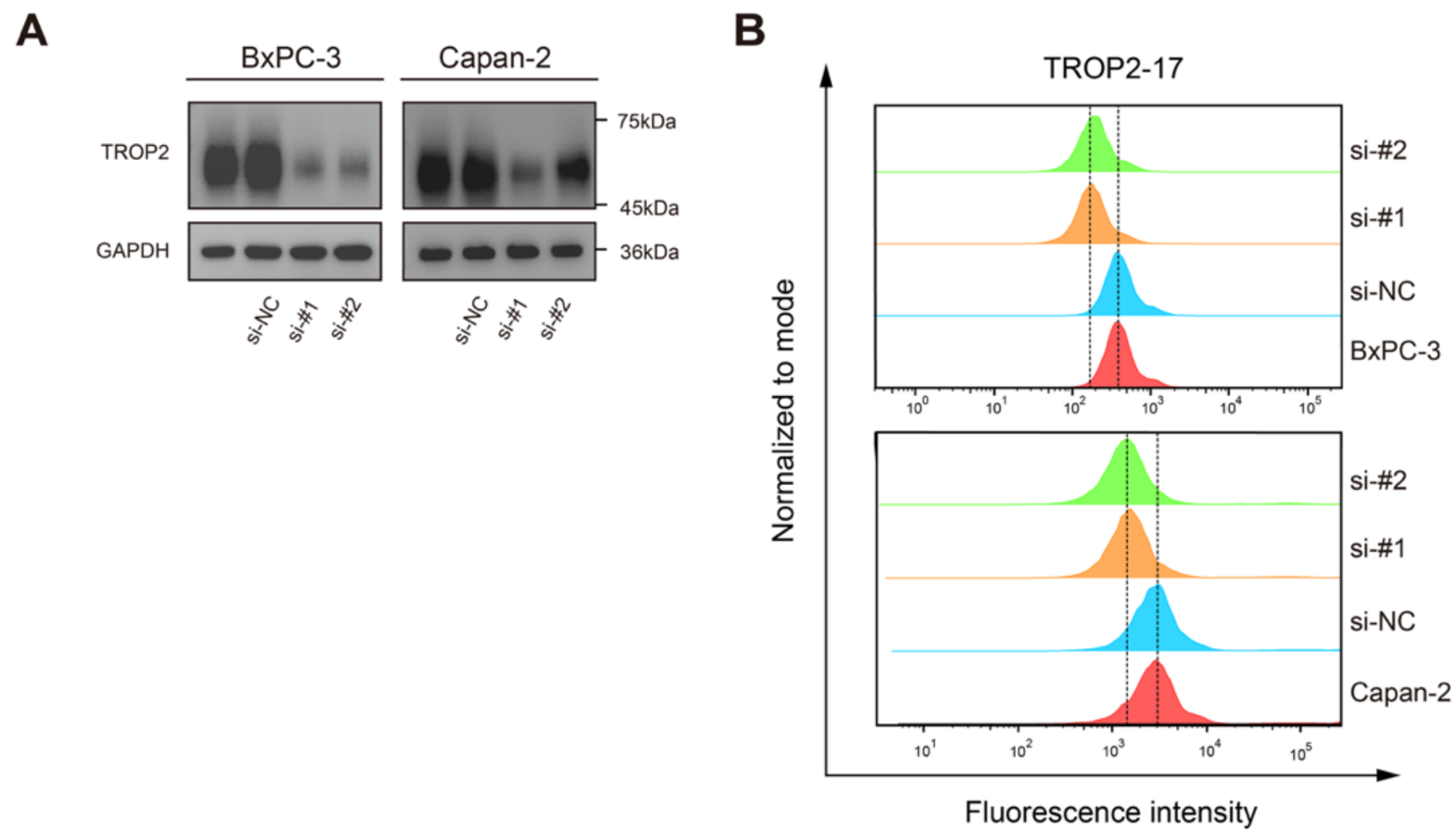

Fig. S3. TROP2 knockdown diminishes TROP2-17 aptamer binding. (A) Western blot showing siRNA mediated knockdown of TROP2 in BxPC-3 and Capan-2 cells (si-#1, si-#2) compared with si-NC. **(B)** Flow cytometry analysis showing reduced binding of Cy5-labeled T17 aptamer to BxPC-3 and Capan-2 cells following TROP2 knockdown.

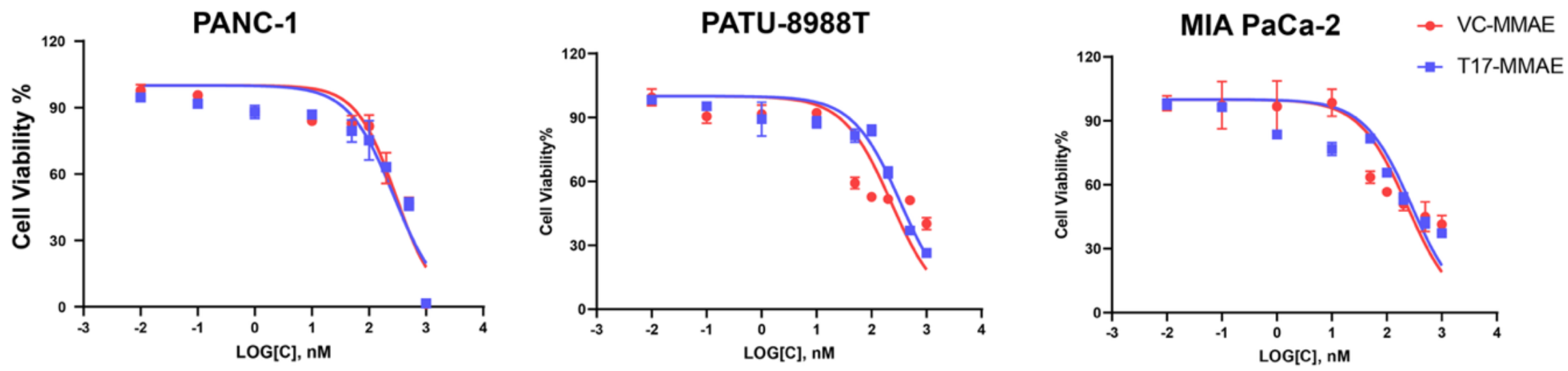

Fig. S4. Dose–response curves of VC–MMAE versus T17-M in PANC-1, PATU-8988T and MIA PaCa-2 cells measured by CCK-8 assay. Cells were treated with the indicated concentrations of VC-MMAE or T17-M for 72 h, and cell viability was normalized to untreated controls. Data are presented as mean ± SD from three independent experiments.

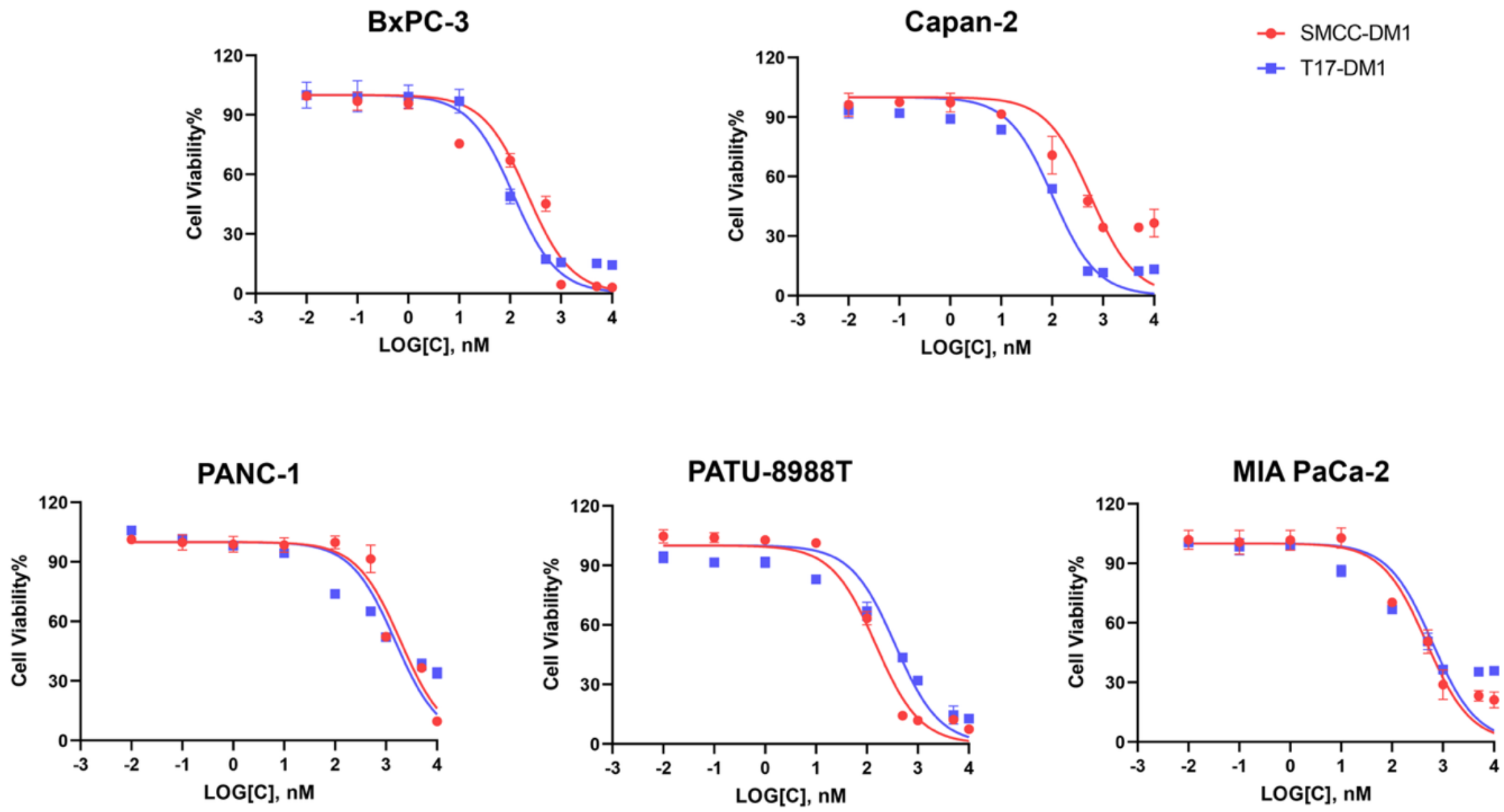

Fig. S5. Dose–response curves of SMCC-DM1 versus T17-DM1 in BxPC-3, Capan-2, PANC-1, PATU-8988T and MIA PaCa-2 cells measured by CCK-8. Cells were treated with serial concentrations of SMCC-DM1 or T17-DM1 for 72 h, and cell viability was assessed by CCK-8 assay. Data are presented as mean ± SD from three independent experiments.

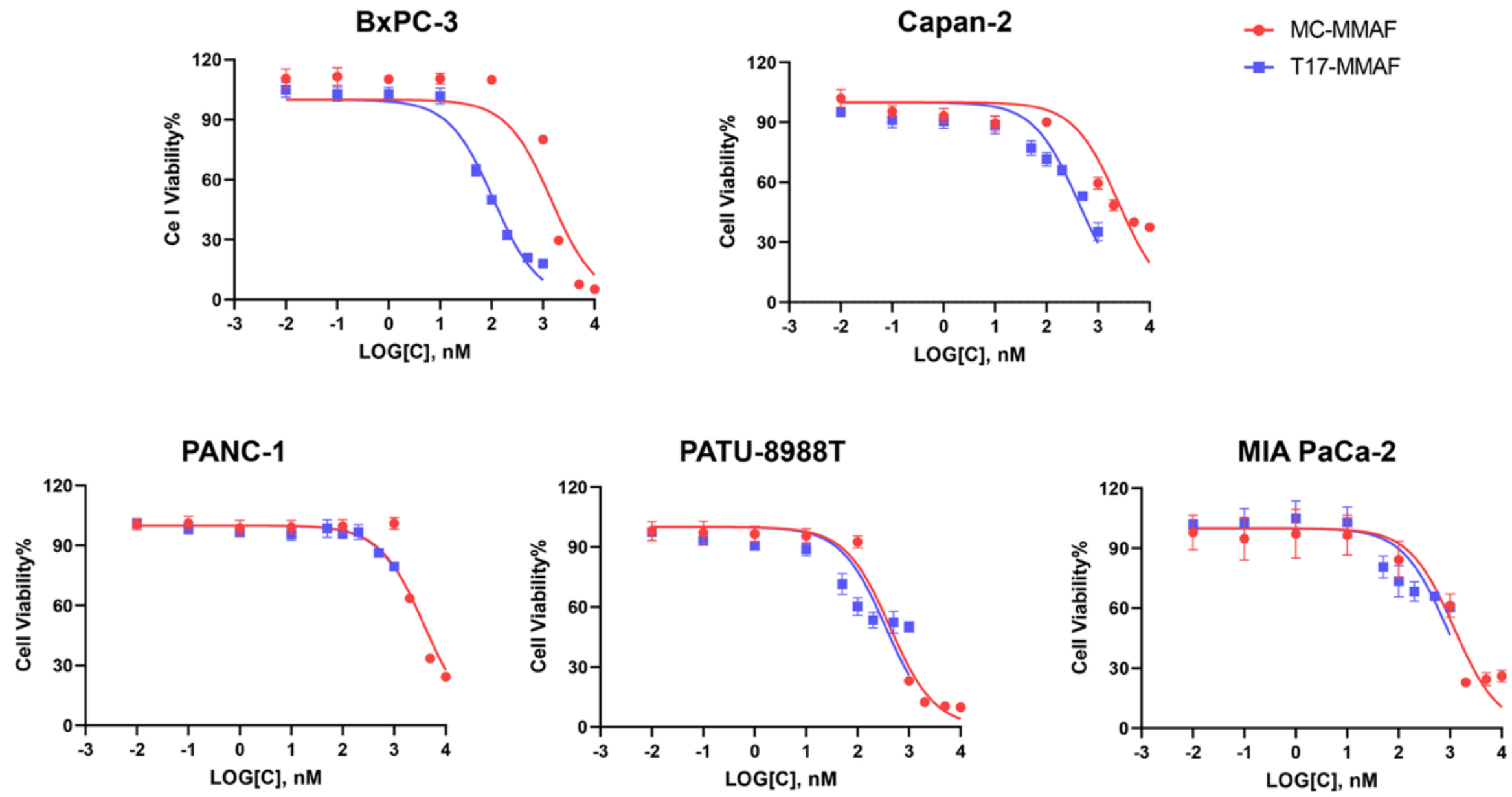

Fig. S6. Dose–response curves of MC–MMAF versus T17-MMAF in BxPC-3, Capan-2, PANC-1, PATU-8988T and MIA PaCa-2 cells measured by CCK-8. Cells were treated with serial concentrations of MC–MMAF or T17–MMAF for 72 h, and cell viability was determined using the CCK-8 assay. Data are presented as mean ± SD from three independent experiments.

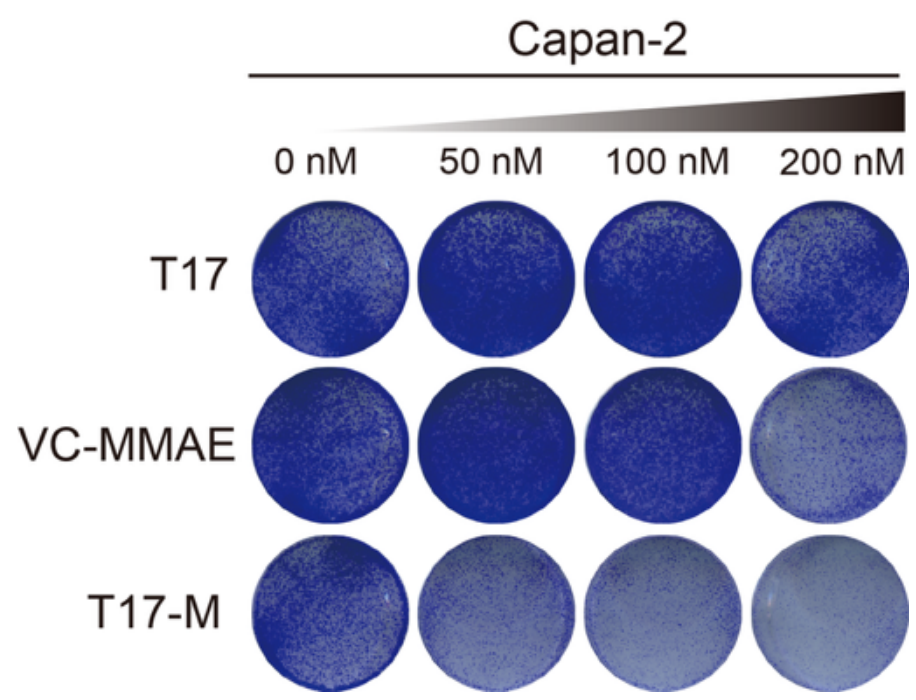

Fig. S7. Long-term cell proliferation assay (stained by crystal violet) of Capan-2 after the indicated treatments and concentrations.

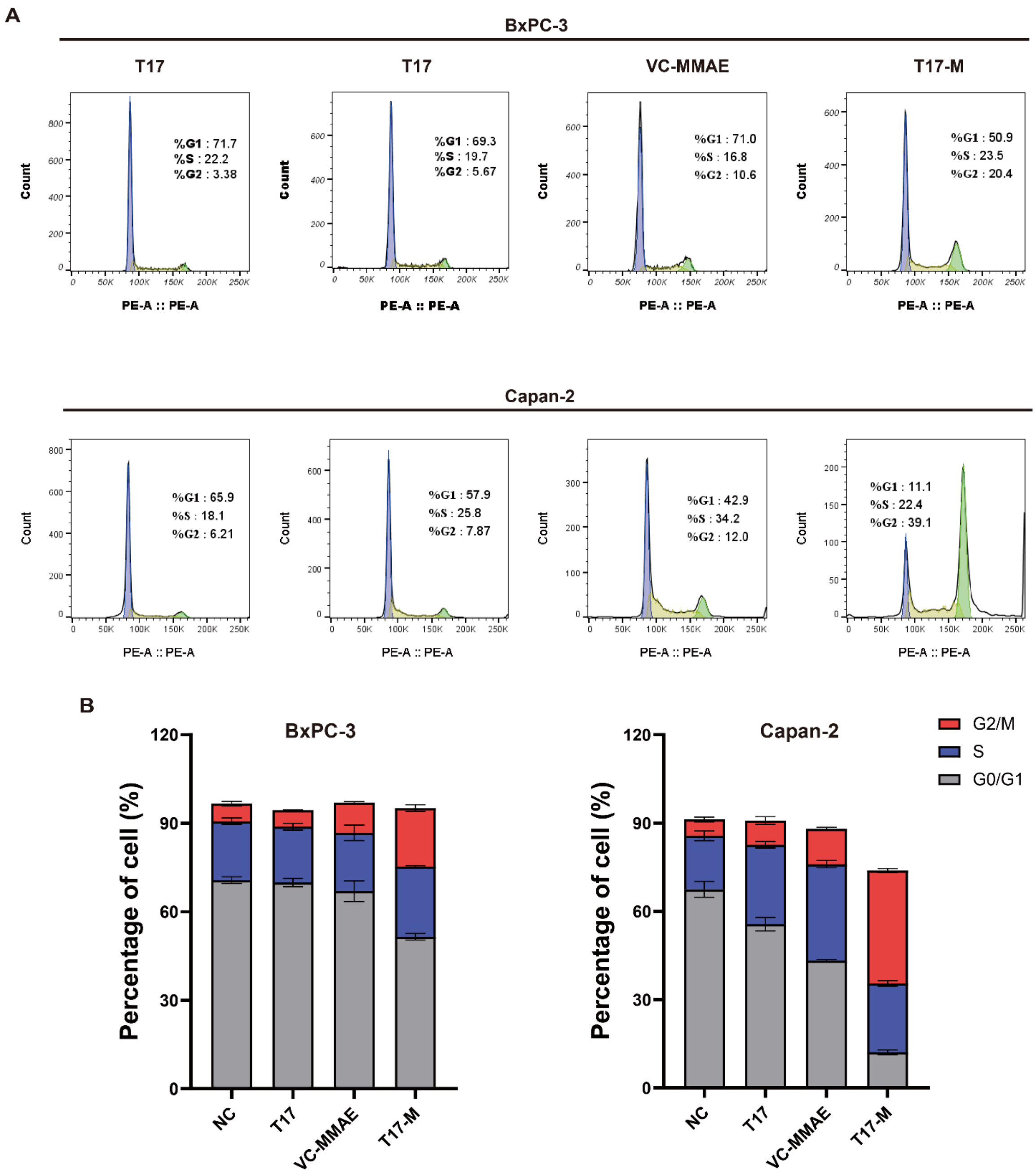

Fig. S8. T17-M induces G2/M cell-cycle accumulation in PDAC cells. (A) Representative cell-cycle histograms for BxPC-3 and Capan-2 cells after indicated treatments. **(B)** Quantification of cell-cycle phase distribution for BxPC-3 (left) and Capan-2 (right). Data are presented as means ± SD from three independent experiments.

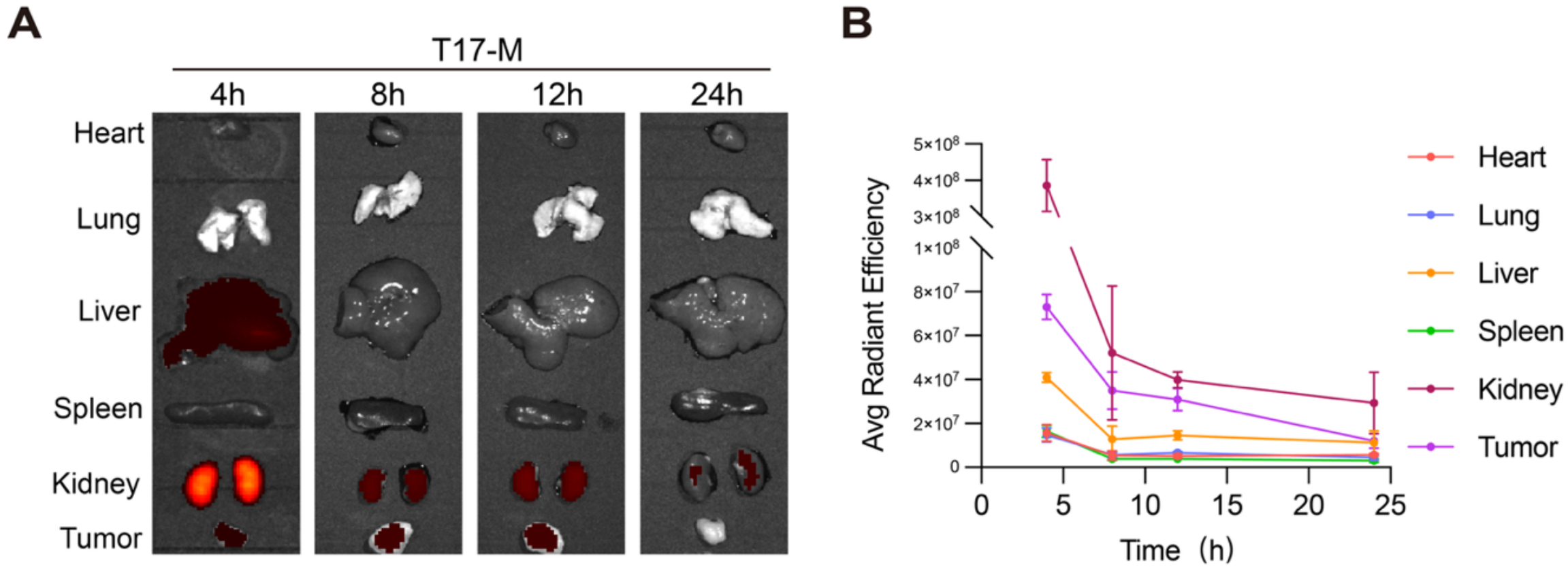

Fig. S9. Biodistribution of T17-M in a Capan-2 CDX model. (A) Ex vivo fluorescence images showing time-resolved biodistribution of T17-M-Cy5 in tumors and major organs (heart, lung, liver, spleen, kidney) of Capan2 CDX mice at 4, 8, 12 and 24 h post-injection. Color bar indicates radiant efficiency. **(B)** Quantification of average radiant efficiency over time in tumors and the indicated organs. Data are presented as means ± SD (n = 3 mice per time point).

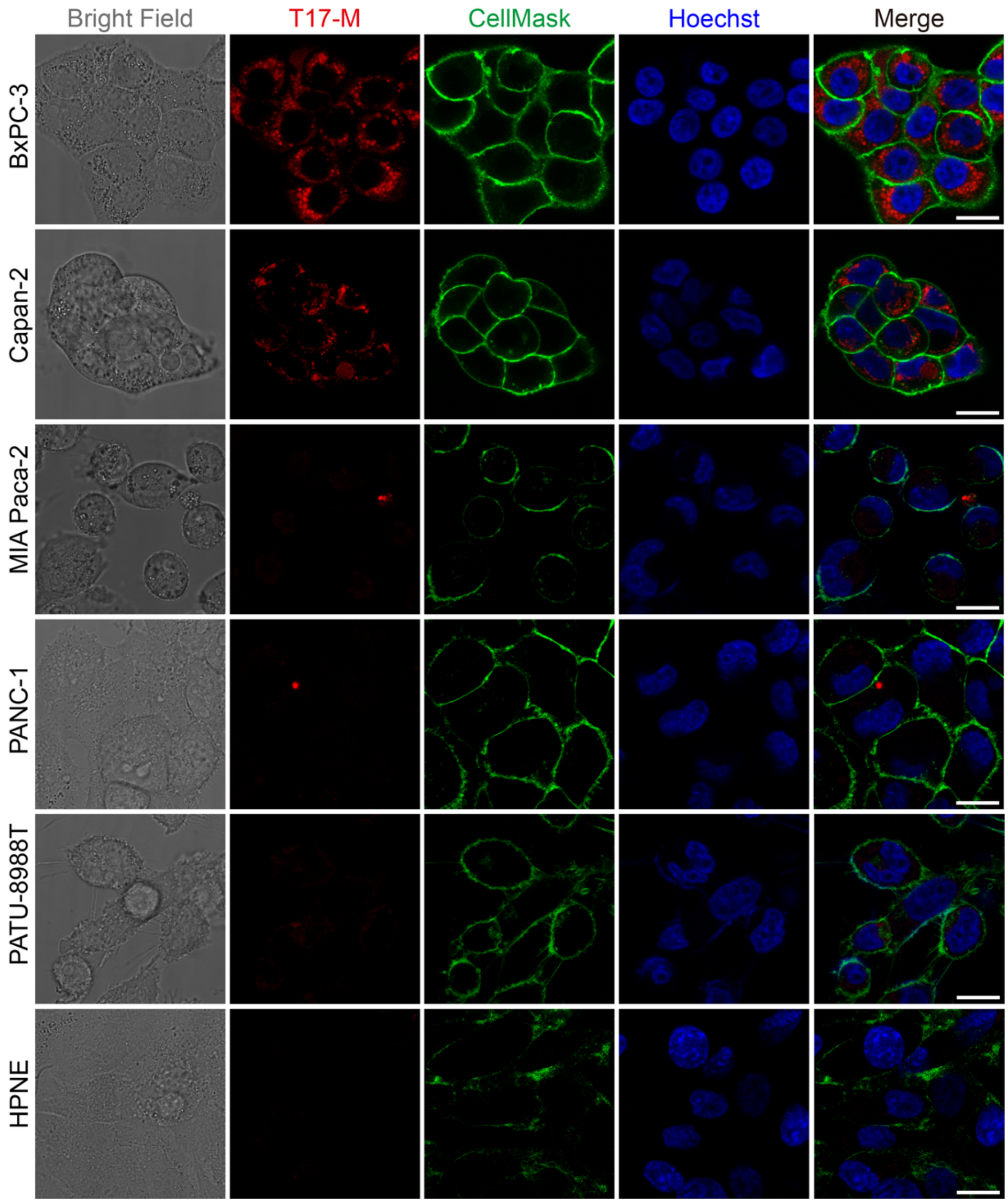

Fig. S10. The internalization of T17-M in different cell lines. Representative images of BxPC-3, Capan-2, MIA PaCa-2, PANC-1, PATU-8988T, and HPNE cells incubated with Cy5-labeled T17-M (red). Plasma membranes were stained with CellMask (green) and nuclei with Hoechst (blue).

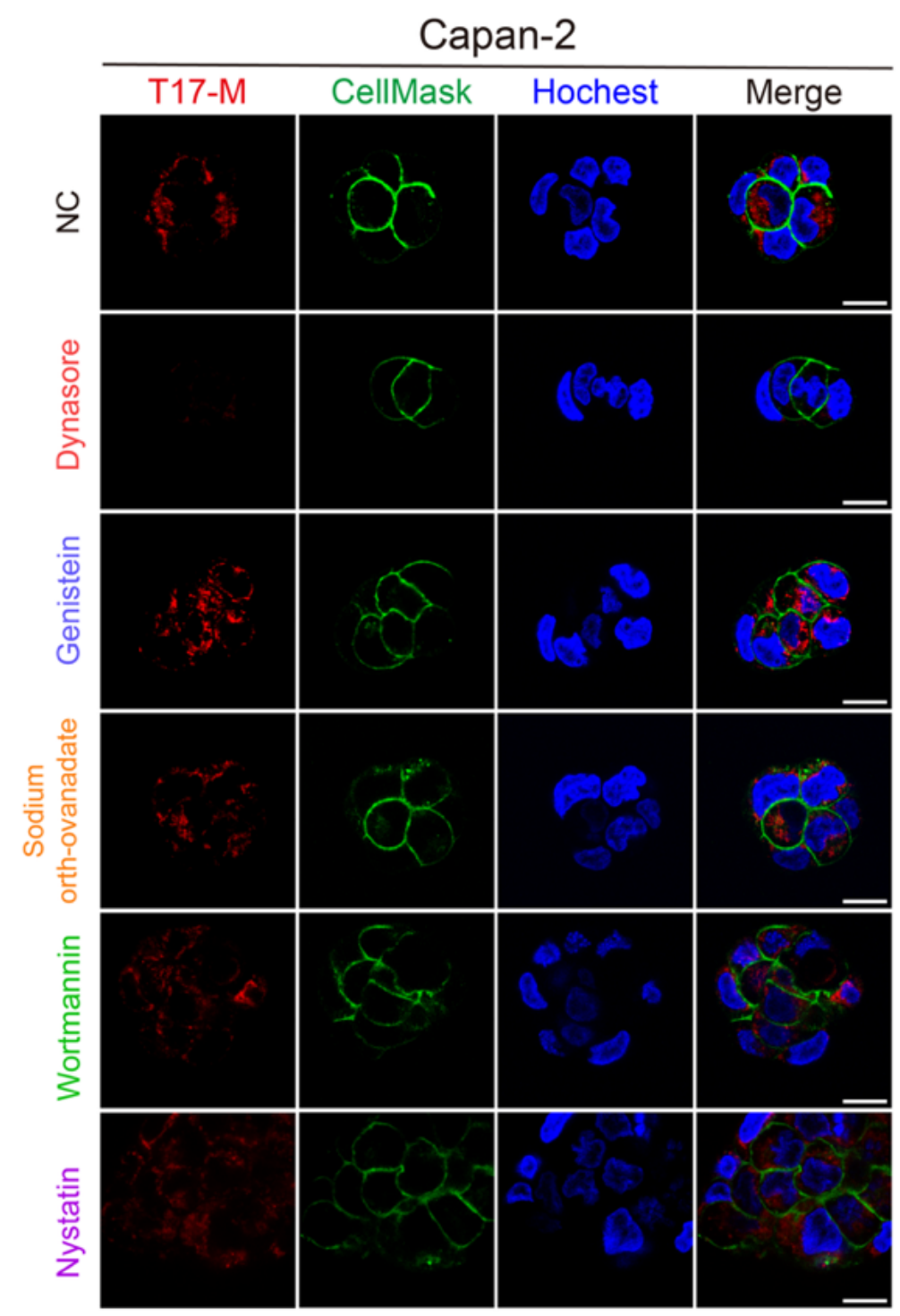

Fig. S11. Effects of endocytic inhibitors on T17-M uptake in Capan-2 cells. Cells were pretreated with dynasore, genistein, sodium orthovanadate, wortmannin or nystatin.

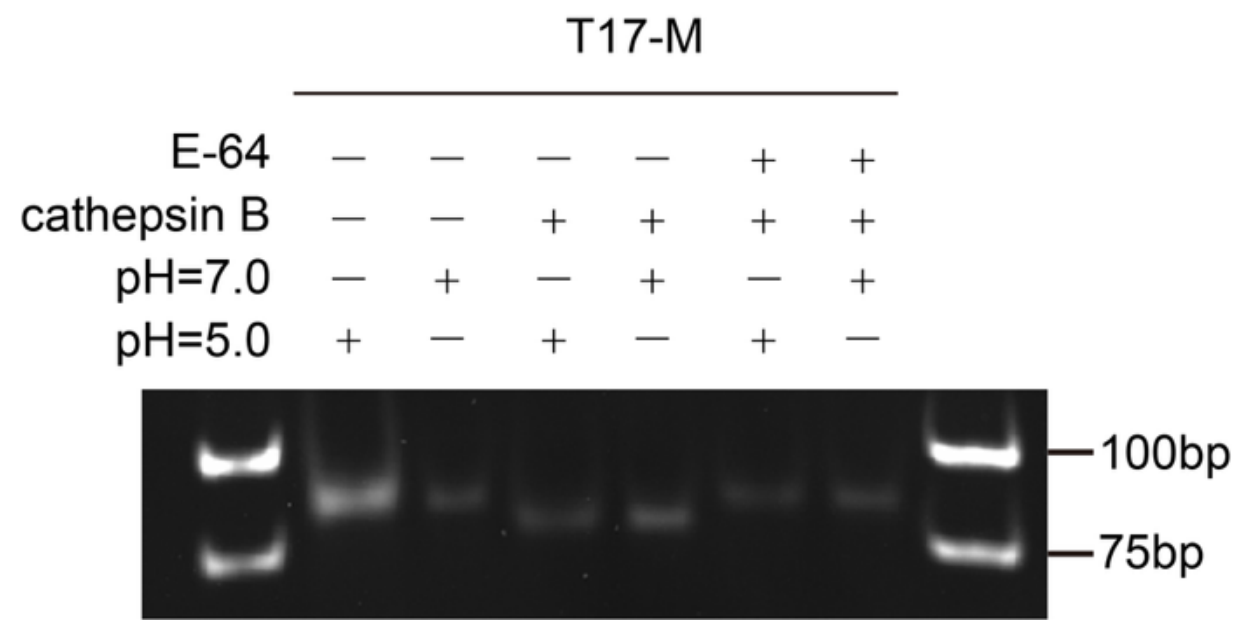

Fig. S12. Cathepsin B and pH dependent cleavage of T17-M. T17-M was incubated under the indicated conditions including cathepsin B, E-64 (broad cysteine-protease inhibitor), and buffer at pH 7.0 or pH 5.0 (simulating the acidic environment of lysosomes). PAGE analysis showing nucleic-acid bands at different position.

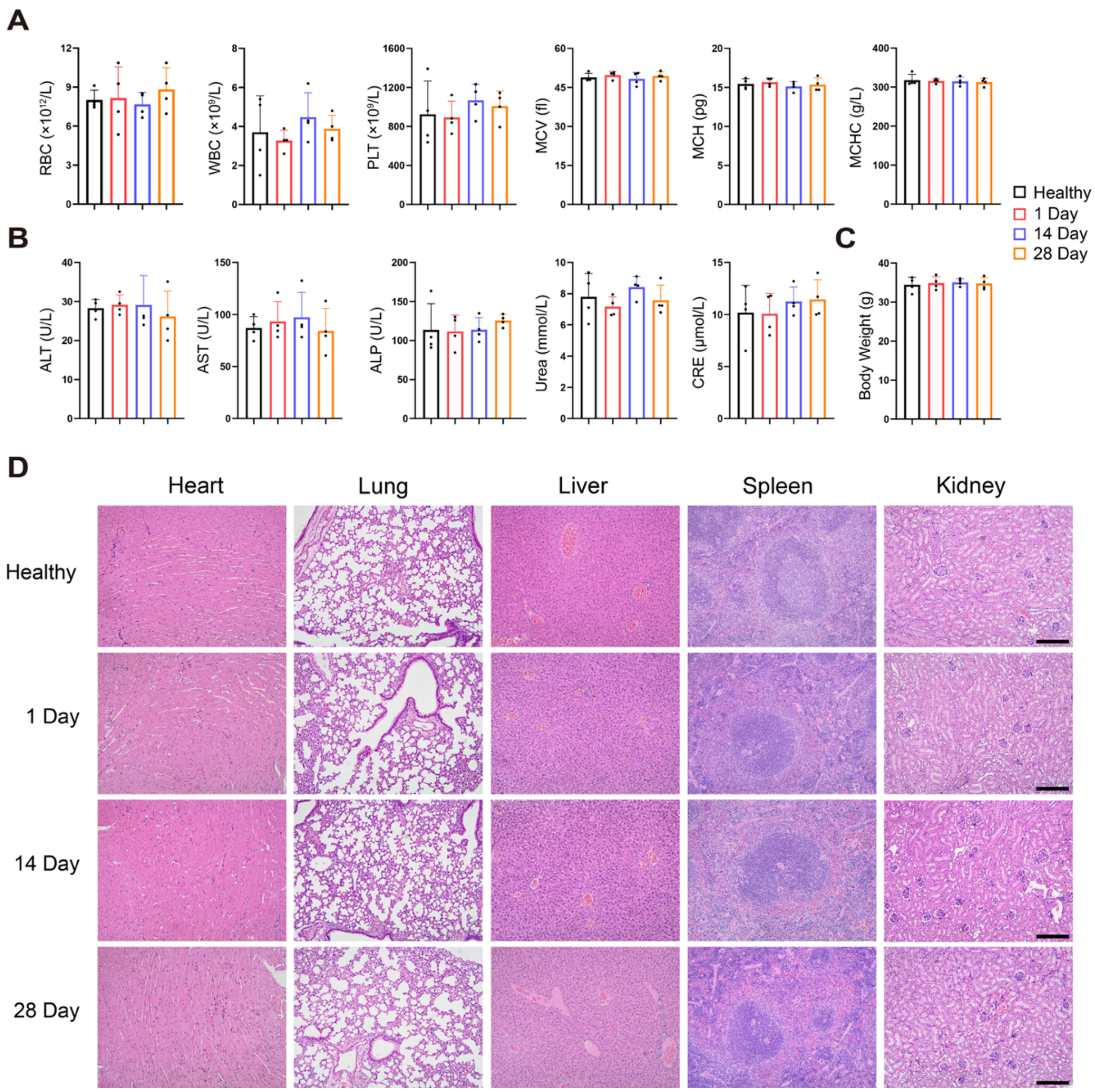

Fig. S13. Systemic safety assessment of T17-M in ICR mice. (A-C) Complete blood counts (RBC, WBC, PLT, MCV, MCH, MCHC), serum biochemistry (ALT, AST, ALP, urea, creatinine), and end-point body weight measured at 1, 14, and 28 days after intravenous T17-M, compared with untreated healthy controls. **(D)** Representative H&E sections of heart, lung, liver, spleen, and kidney at the indicated time points showing no overt histopathological abnormalities after T17-M treatment. Scale bars, 100 µm. Data in **(A to C)** are presented as means ± SD (n = 4 mice per group). Statistical significance was assessed using one-way ANOVA followed by Dunnett's multiple-comparisons test, with each T17-M time-point group compared with the healthy control group. No statistically significant differences were detected.

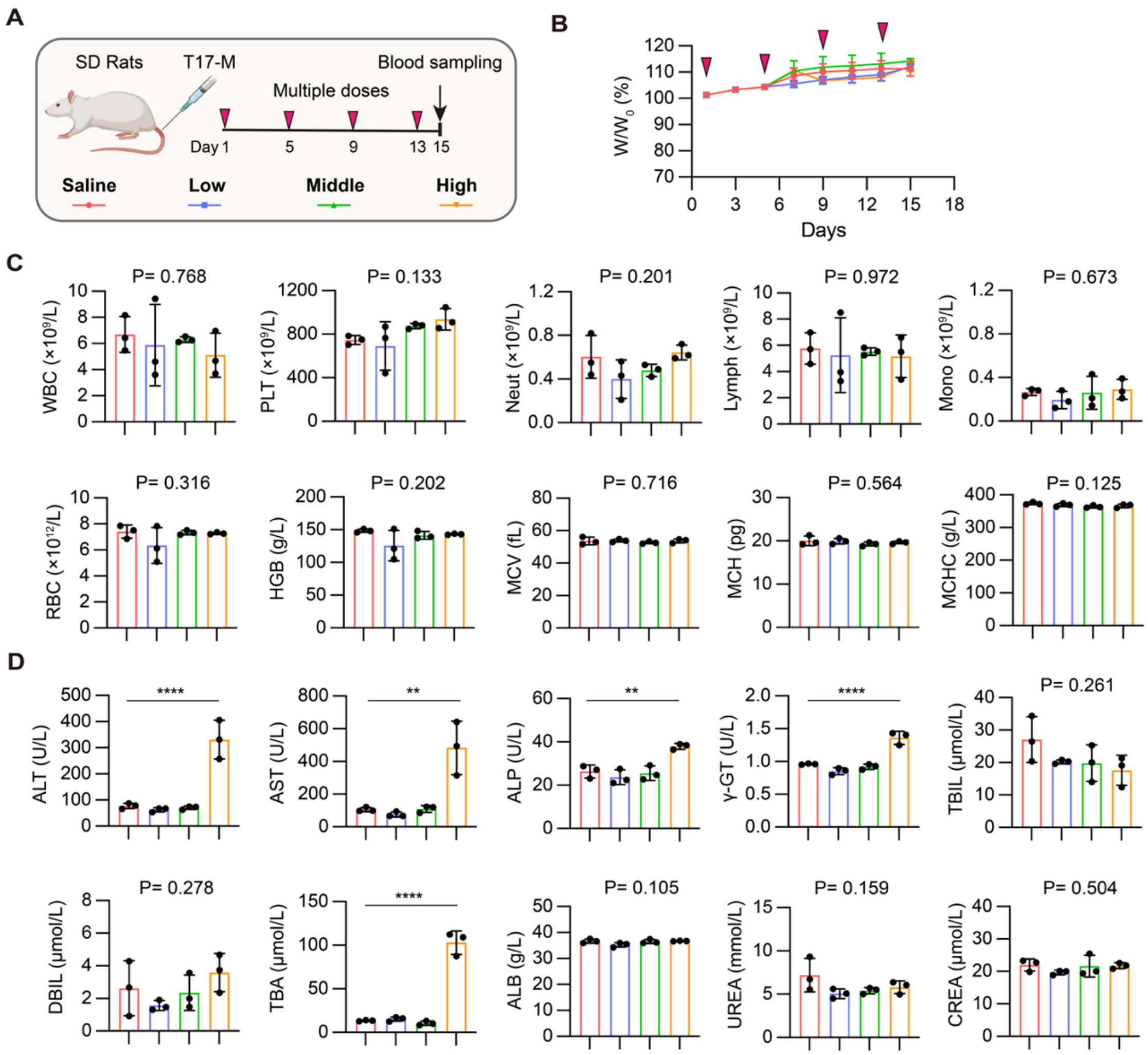

Fig. S14. Repeated-dose systemic safety evaluation of T17-M in Sprague–Dawley rats. **(A)** Experimental scheme for the repeated-dose safety study. Sprague–Dawley rats received intravenous administration of saline or low, middle, or high doses of T17-M on days 1, 5, 9, and 13, followed by blood collection on day 15. The figure was created using BioRender (https://BioRender.com/taiu5da). **(B)** Body weight changes normalized to the corresponding baseline values (W/W_0). **(C)** Hematologic parameters, including white blood cell count (WBC), platelet count (PLT), neutrophil count (Neut), lymphocyte count (Lymph), monocyte count (Mono), red blood cell count (RBC), hemoglobin concentration (HGB), mean corpuscular volume (MCV), mean corpuscular hemoglobin (MCH), and mean corpuscular hemoglobin concentration (MCHC). **(D)** Serum biochemical parameters, including alanine aminotransferase (ALT), aspartate aminotransferase (AST), alkaline phosphatase (ALP), γ-glutamyl transferase (γ-GT), total bilirubin (TBIL), direct bilirubin (DBIL), total bile acids (TBA), albumin (ALB), urea, and creatinine (CREA). Data are presented as means ± SD (n = 3 rats per

group). Hematologic and serum biochemical parameters in **(C)** and **(D)** were analyzed using one-way ANOVA followed by Dunnett's multiple-comparisons test, with each T17-M dose group compared with the saline control. Exact overall P values are shown where indicated, and adjusted P values for the indicated pairwise comparisons are denoted by asterisks. *$P < 0.05$, **$P < 0.01$, ***$P < 0.001$, and ****$P < 0.0001$.

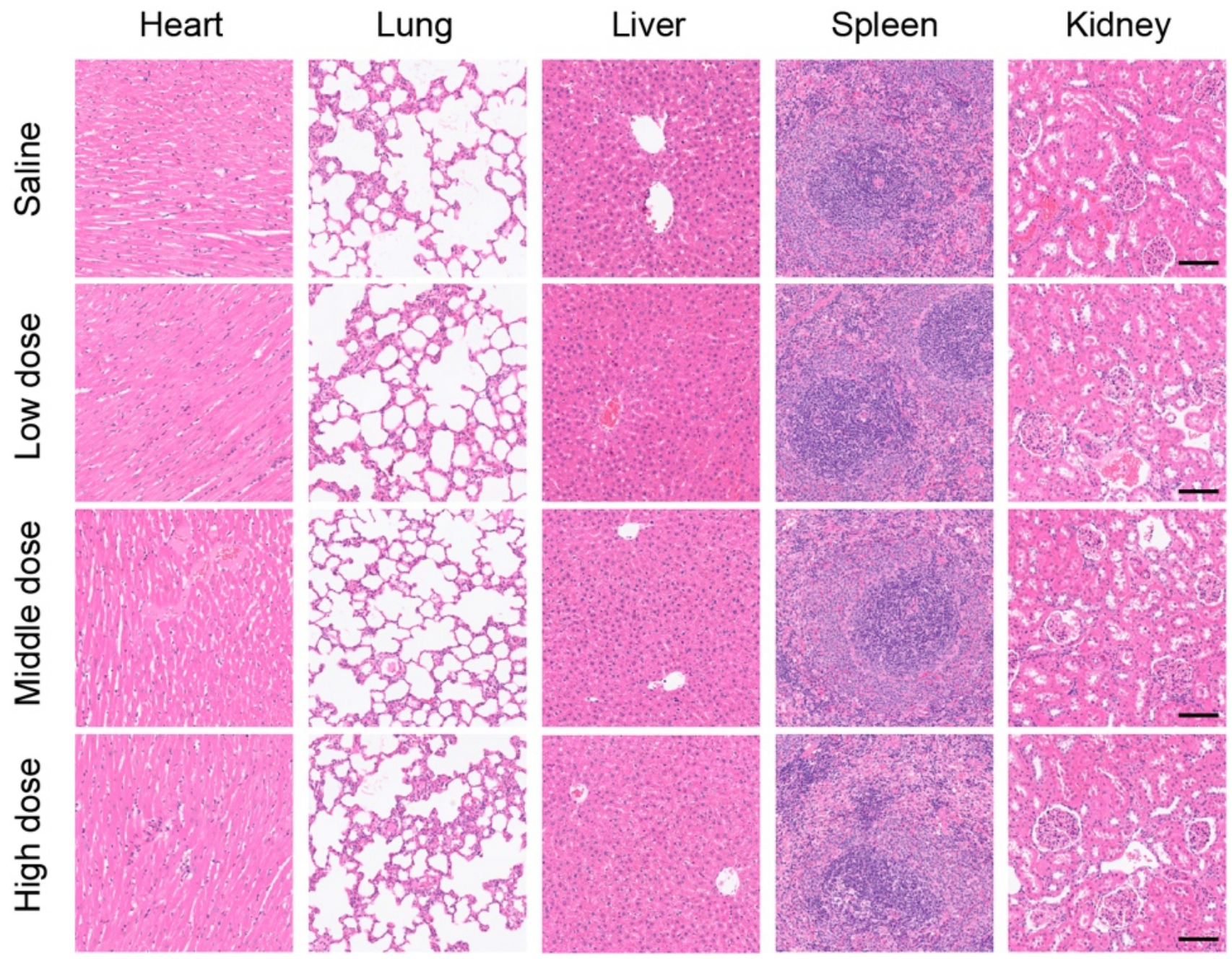

Fig. S15. Representative H&E sections of heart, lung, liver, spleen, and kidney from rats treated with saline or low, middle, or high doses of T17-M. Scale bars, 100 µm.

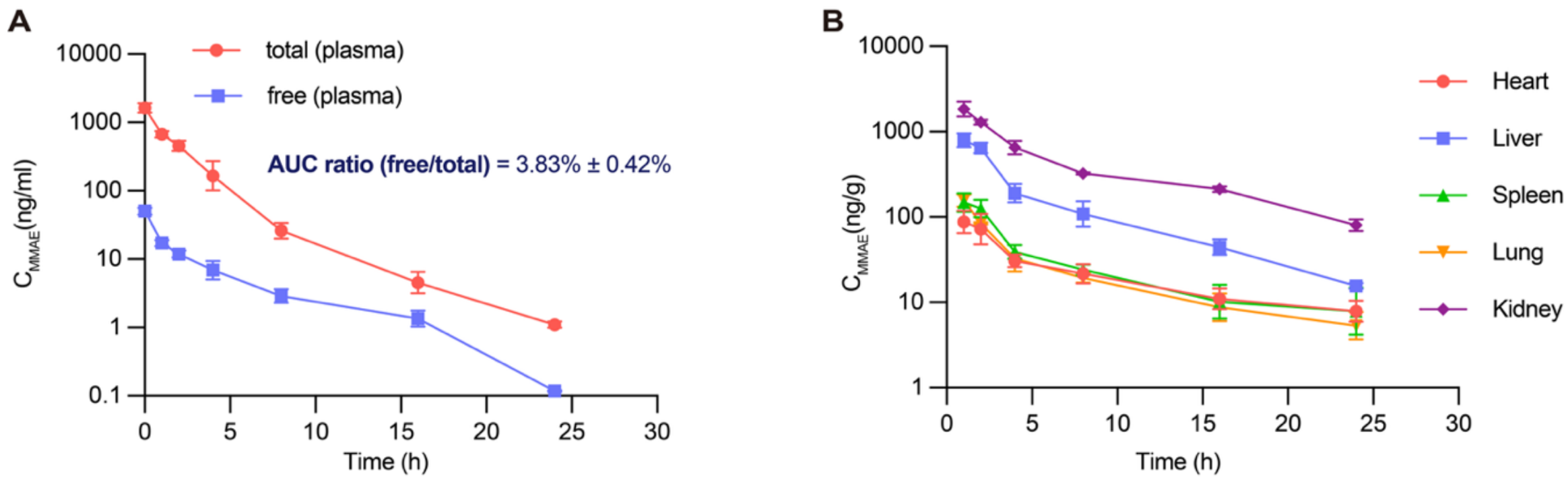

Fig.S16. Pharmacokinetic and tissue-distribution profiles of T17-M in Sprague–Dawley rats. (A) Plasma concentration–time profiles of total and free MMAE following intravenous administration of T17-M. The free to total plasma AUC ratio was calculated individually for each rat and is presented as mean ± SD (3.83 ± 0.42%). (B) MMAE concentration time profiles in the heart, liver, spleen, lung, and kidney following T17-M administration. Data in (A) are presented as means ± SD (n = 3 rats), and data in (B) are presented as means ± SD (n = 3 rats per time point). Pharmacokinetic parameters were calculated by noncompartmental analysis.

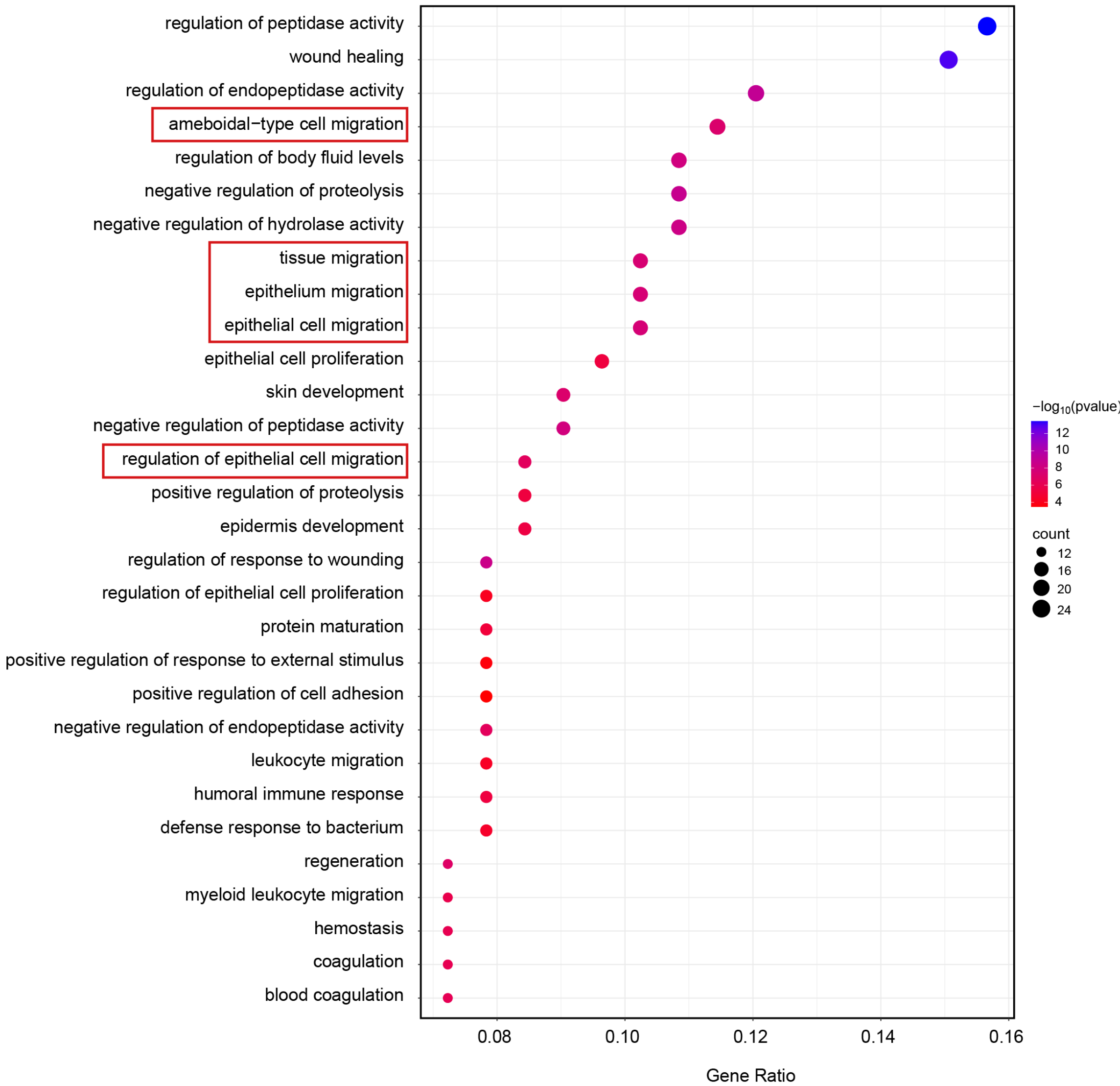

Fig. S17. GO enrichment analysis (Biological Process) for TACSTD2 in PDAC. Red boxes highlight terms related to tumor cell metastasis.

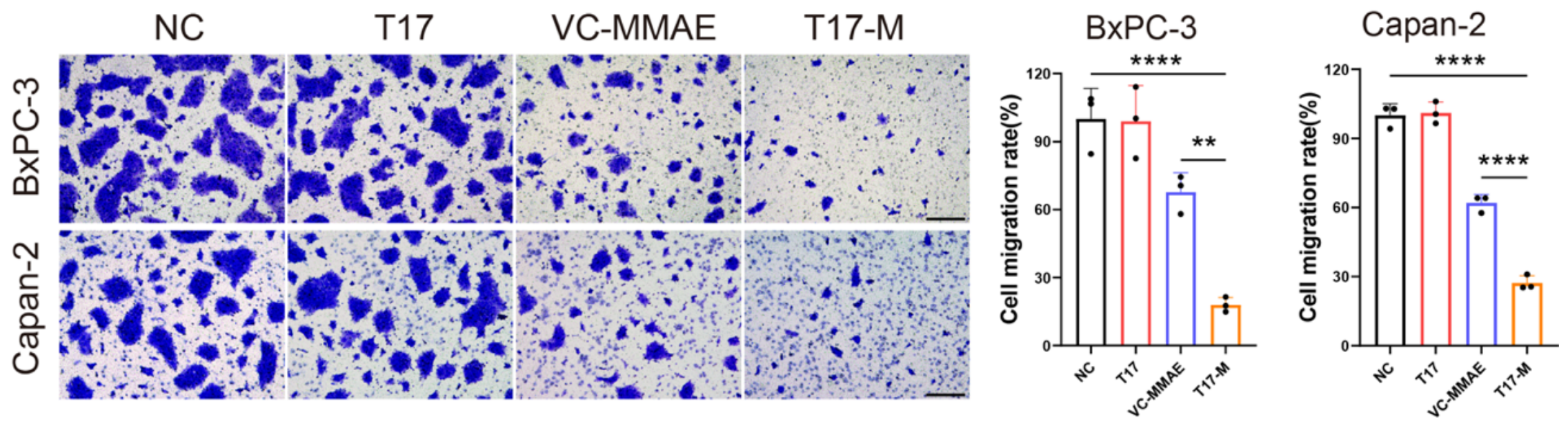

Fig. S18. Transwell migration assays showing that T17-M suppresses cell migration in BxPC-3 and Capan-2 cells compared with NC, T17, and VC-MMAE. Representative crystal-violet images (left) and quantification of migration rate (right; mean ± SD, n = 3). Scale bar, 200 µm. Data are presented as means ± SD from three independent experiments. Statistical significance was assessed separately for each cell line using one-way ANOVA followed by Tukey's multiple-comparisons test. Selected pairwise comparisons are shown. Scale bar, 200 µm. ns, not significant; **P < 0.01 and ****P < 0.0001.

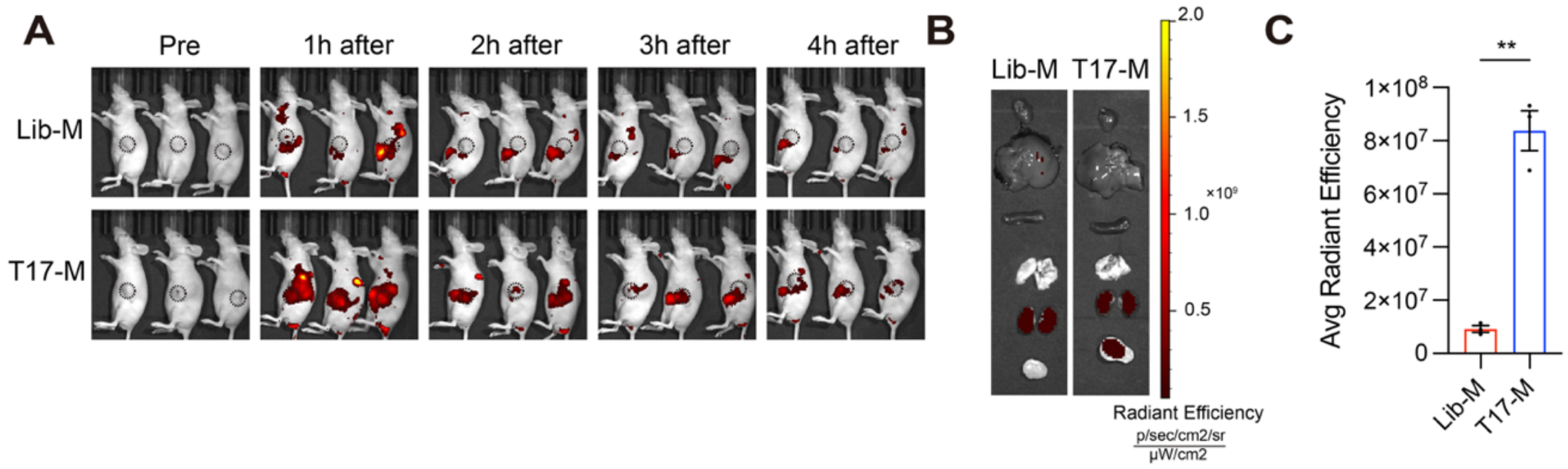

Fig. S19. Tumor targeting of T17-M versus a library control. (A) In vivo fluorescence images showing whole-body fluorescence after injection of Cy5-labeled Lib-M or T17-M at baseline (Pre) and 1, 2, 3, and 4h post-injection. Color bars indicate radiant efficiency. **(B)** Ex vivo fluorescence images of major organs and tumors 3 hours after injection of Lib-M or T17-M. **(C)** Quantification of average radiant efficiency in tumors. Data are presented as means ± SD (n = 3 mice per group). Statistical significance was determined using a two-tailed unpaired Student's t test. **P < 0.01.

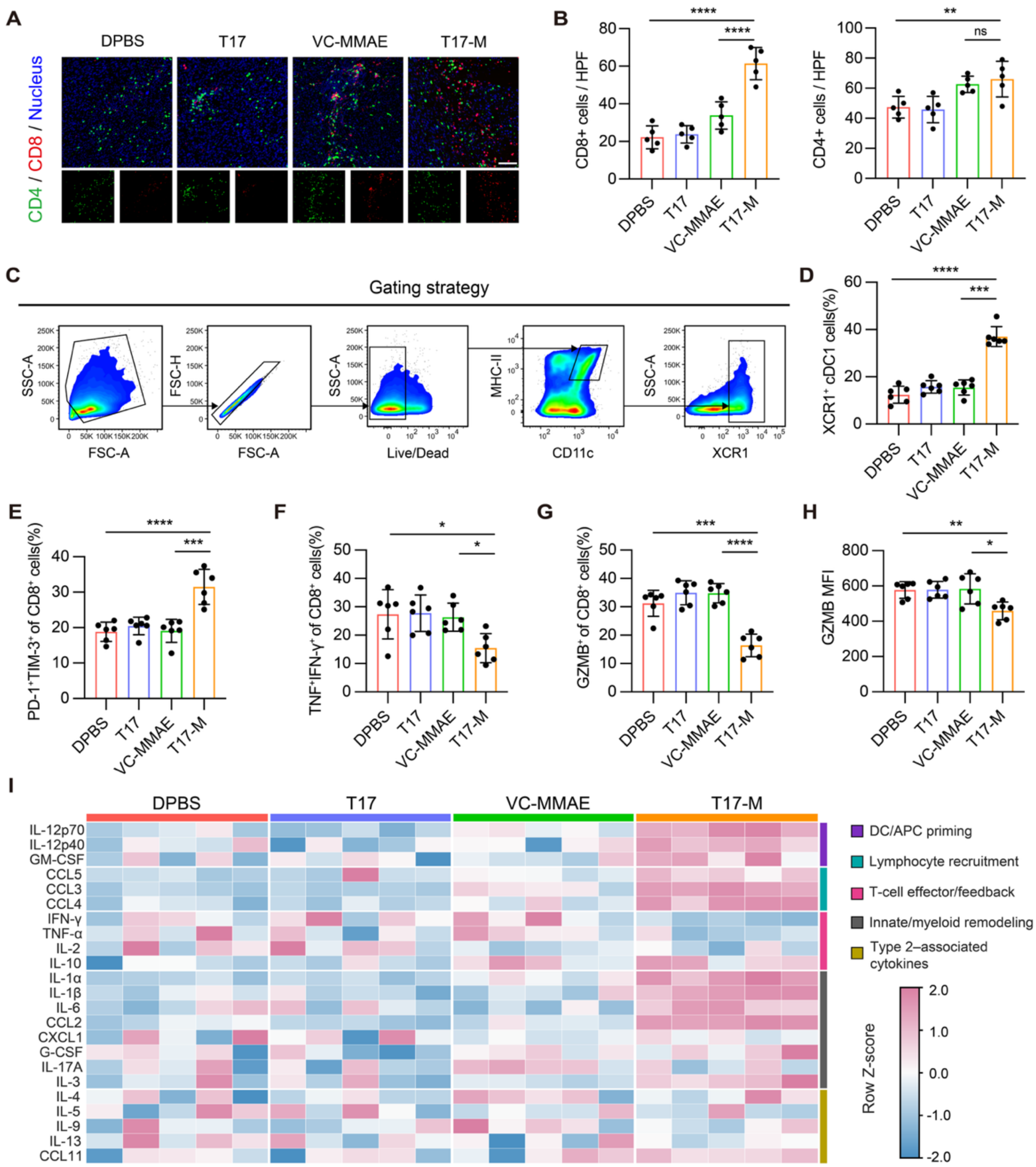

Fig. S20. T17-M promotes immune activation and reshapes the tumor immune microenvironment. (A) Multiplex IHC of orthotopic tumors showing CD8 (red) and CD4 (green) T cell infiltration after the indicated treatments. Nuclei were stained by DAPI (blue). Scale bar, 100 µm. **(B)** Quantification of $CD8^+$ (left) and $CD4^+$ (right) cells per high-power field (HPF). **(C)** Representative flow cytometry gating strategy. **(D)** Quantification of $XCR1^+CD11c^+$ activated dendritic cells in tumors following the indicated treatments. **(E)** Flow cytometric quantification of PD-1^+TIM-3^+ exhausted $CD8^+$ T cells among tumor-infiltrating $CD8^+$ T cells. **(F)** Quantification of TNF-

α^+IFN-γ^+ polyfunctional CD8$^+$ T cells following stimulation. **(G and H)** Flow cytometric analysis of granzyme B expression in CD8$^+$ T cells, including the percentage of GZMB$^+$ CD8$^+$ T cells **(G)** and GZMB median fluorescence intensity (MFI) **(H)**. **(I)** Heatmap showing tumor cytokine profiles across treatment groups. Cytokines were categorized according to their functional annotation, including DC/APC priming, lymphocyte recruitment, T cell effector responses, innate/myeloid remodeling, and type 2–associated cytokines. Data in (B) are presented as means ± SD (n = 5 mice per group), data in (D to H) are presented as means ± SD (n = 6 mice per group). Panels (A) and (I) include samples from 5 mice per group. Statistical significance in (B) and (D to H) was assessed separately using ordinary one-way ANOVA followed by Tukey's multiple-comparisons test. Selected pairwise comparisons are shown. ns, not significant; *$P < 0.05$, **$P < 0.01$, ***$P < 0.001$, and ****$P < 0.0001$.

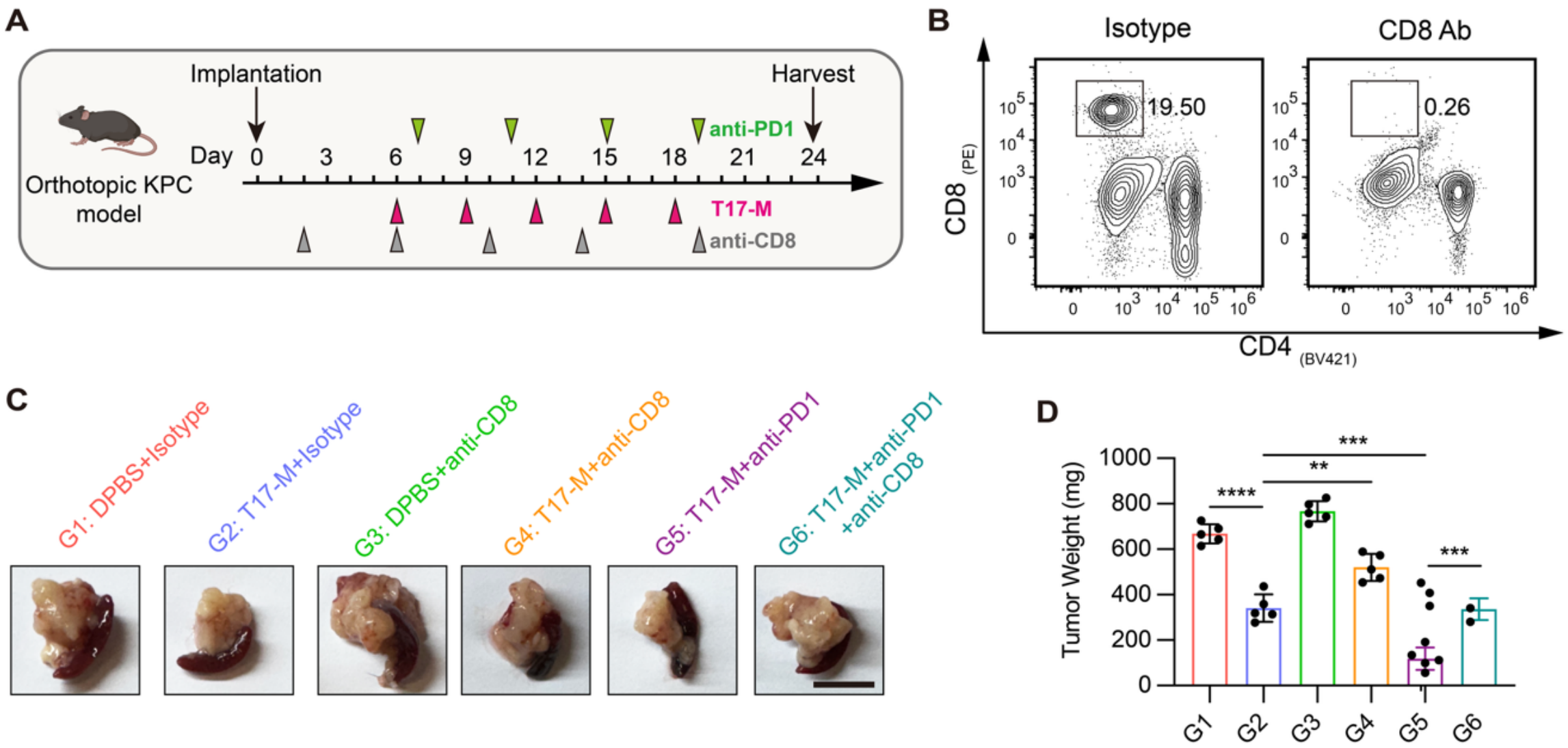

Fig. S21. CD8⁺ T cell depletion attenuates the antitumor activity of T17-M and its combination with PD-1 blockade. (A) Experimental scheme for the $CD8^+$ T cell depletion study in the KPC-derived syngeneic orthotopic PDAC model. Mice received T17-M, anti-PD1 antibody, anti-CD8 antibody, or the corresponding isotype controls at the indicated time points, and tumors were collected on day 24. The figure was created using BioRender (https://BioRender.com/6kqedug). **(B)** Representative flow cytometry plots confirming depletion of $CD8^+$ T cells following anti-CD8 antibody treatment compared with the isotype control. Numbers indicate the percentages of $CD8^+$ T cells. **(C)** Representative images of excised orthotopic tumors from the indicated treatment groups. Scale bar, 1 cm. **(D)** Endpoint tumor weights in the indicated groups. Data are presented as means ± SD (n = 5 mice per group**).** Statistical significance in **(D)** was assessed using ordinary one-way ANOVA followed by Tukey's multiple-comparisons test. Selected pairwise comparisons are shown. **P < 0.01, ***P < 0.001, and ****P < 0.0001.

Table S1

Aptamer	Sequence	Length
TROP2-1	CTACGGTGCCTTGAAGTGACTCGTTCCGTCCCTGGCCGCTCCTACTAT CGATGTGTCATAGCAGGTCACTTCCAGG	76
TROP2-17	CTACGGTGCCTTGAAGTGACTCGACACCCTCGTCCCCGGCCGCTCCTG CTCAATTTCATAGCAGGTCACTTCCAGG	76

Table S2

Aptamer	Van der Waal (KJ/mol)	Electrostatic (KJ/mol)	Polar solvation (KJ/mol)	Non-polar solvation (KJ/mol)	Binding energy (KJ/mol)
TROP2-1	-31.06±0.49	-8.59±0.27	5.59±0.62	-3.58±0.17	-37.64±1.09
TROP2-17	-46.95±1.09	-6.72±0.35	5.24±0.49	-7.96±0.33	-56.39±1.26
